# Investigation of acidic free-glycans in urine and their alteration in cancer

**DOI:** 10.1093/glycob/cwaa100

**Published:** 2020-10-29

**Authors:** Ken Hanzawa, Miki Tanaka-Okamoto, Hiroko Murakami, Mikio Mukai, Hidenori Takahashi, Takeshi Omori, Kenji Ikezawa, Kazuyoshi Ohkawa, Masayuki Ohue, Yasuhide Miyamoto

**Affiliations:** Department of Molecular Biology, Osaka International Cancer Institute, 3-1-69 Otemae, Chuo-ku, Osaka 541-8567, Japan; Department of Molecular Biology, Osaka International Cancer Institute, 3-1-69 Otemae, Chuo-ku, Osaka 541-8567, Japan; Department of Molecular Biology, Osaka International Cancer Institute, 3-1-69 Otemae, Chuo-ku, Osaka 541-8567, Japan; Department of Medical Checkup, Osaka International Cancer Institute, 3-1-69 Otemae, Chuo-ku, Osaka 541-8567, Japan; Department of Gastroenterological Surgery, Osaka International Cancer Institute, 3-1-69 Otemae, Chuo-ku, Osaka 541-8567, Japan; Department of Gastroenterological Surgery, Osaka International Cancer Institute, 3-1-69 Otemae, Chuo-ku, Osaka 541-8567, Japan; Department of Hepatobiliary and Pancreatic Oncology, Osaka International Cancer Institute, 3-1-69 Otemae, Chuo-ku, Osaka 541-8567, Japan; Department of Hepatobiliary and Pancreatic Oncology, Osaka International Cancer Institute, 3-1-69 Otemae, Chuo-ku, Osaka 541-8567, Japan; Department of Gastroenterological Surgery, Osaka International Cancer Institute, 3-1-69 Otemae, Chuo-ku, Osaka 541-8567, Japan; Department of Molecular Biology, Osaka International Cancer Institute, 3-1-69 Otemae, Chuo-ku, Osaka 541-8567, Japan

**Keywords:** cancer, free glycan, HPLC, SRM, urine

## Abstract

Alterations to glycans in cancer patients have been used to identify novel tumor biomarkers. Most of these studies have focused on protein glycosylation but less attention has been paid to free-glycans. Here, we analyzed acidic free-glycans in the urine of cancer patients to identify novel tumor marker candidates. Specifically, urine samples were collected from patients with gastric cancer, pancreatic cancer and cholangiocarcinoma as well as normal controls. The free-glycans were extracted from creatinine-adjusted urine and fluorescently labeled with 2-aminopyridine. Initially, we performed profiling of urinary free-glycans by high-performance liquid chromatography and mass spectrometry with enzymatic and chemical degradation. More than 100 glycans, including novel structures, were identified. The chromatographic peaks suggested some of these glycans were present at elevated levels in cancer patients. To verify cancer-associated alterations, we compared the glycan levels between cancer patients and normal controls by selected reaction monitoring. Representative structures of glycans with elevated levels in cancer patients included the following: small glycans related to sialyllactose; sialyl Lewis X; lactose- and N-acetyllactosamine (LacNAc) type-II-core glycans with LacNAc (type-I or II)-extensions and modifications of α1,3/4-fucose and/or 6-sulfate on the Glc/GlcNAc; free-*N*-glycans containing sialylation or β1,6-branch of 6-sulfo Lewis X; novel NeuAcα2-3Galβ1-4(+/−Fucα1-3) Xylα1-3Glc glycans. Our results provide further insight into urinary free-glycans and suggest the potential utility of these compounds as tumor markers.

## Introduction

Tumor markers play an important role in the clinical diagnosis of cancer, as well as for assessing the effectiveness of treatment and monitoring possible recurrence of the disease. Some tumor markers are based on serum glycan antigens, which are present at elevated levels in certain types of cancer. Examples of such serum glycans include CA19-9, sialyl Tn, sialyl Lewis X and Du-Pan-2, which can be detected using monoclonal antibodies. However, there are some patients whose existing marker levels do not increase despite their cancer progression. To improve diagnosis, further development of novel markers is urgently needed. In recent years, to identify glycan tumor markers, glycoproteins in serum and urine have been actively investigated using various detection methods including HPLC, MS and lectin microarrays, as well as immunochemical techniques (Arnold et al. 2011; Alley Jr. et al. 2012; Biskup et al. 2013; Hua et al. 2013; Gbormittah et al. 2014; Pan et al. 2014; Hamfjord et al. 2015; Ideo et al. 2015; Liang et al. 2015; Varki et al. 2015b; Doherty et al. 2018). Our laboratory previously reported that HPLC-based separation and mass spectrometry-based quantification of serum *O*-glycans showed increased levels of the CA19-9 related glycan with sialyl Lewis A and sialyl Tn in cancer patients (Tanaka-Okamoto et al. 2016, 2018a). In addition, several potential tumor marker candidates were identified by analysis of serum *O*-linked glycans focused on minor acidic structures (Tanaka-Okamoto et al. 2017, 2018b). By contrast with protein glycosylation, few studies have been performed to assess free-glycans as potential tumor markers. The reducing end of these free-glycans is not covalently attached to other molecules. Intriguingly, several studies suggest abnormal accumulation of these free-glycans in cancer. For example, elevated cytosolic levels of sialylated free-*N*-glycans were found in gastric cancer cell lines (Ishizuka et al. 2008). Our laboratory previously reported that cells from pancreatic cancer specimens also showed accumulation of sialylated free-*N*-glycans (Yabu et al. 2013b). Moreover, free-*N*-glycans containing unusual sialic acid, KDN as well as common sialic acid, NeuAc were found in prostate cancer specimens (Yabu et al. 2013a). Taken together, these studies suggested free-glycans that are elevated in cancer patients might be useful as tumor marker candidates. Urine contains abundant amounts of free-glycans that are excreted from the body. Moreover, because samples can be collected noninvasively, urine is an attractive source of such tumor markers. The composition of urinary glycans reflects alterations in glycan metabolism. For example, it have been reported that urinary levels of free-glycans containing a lactose-core, related to the milk oligosaccharides, increased in healthy individuals during pregnancy and lactation or patients with hyperprolactinemia (Hallgren et al. 1977; Ekman et al. 2015). Patients with oligosaccharidosis, a subgroup of lysosomal storage diseases caused by congenital deficiencies of glycan-metabolic enzymes, are known to exhibit an abnormal increase of free-glycans in the body. Urine from these patients contain elevated levels of incompletely degraded free-glycans (Humbel and Collart 1975; Winchester 2005; Bruggink et al. 2012; Xia et al. 2013). Several studies of urine from cancer patients showed raised levels of sialyllactose and sialyl LacNAc (Shimada et al. 1995; Zhang et al. 2013). These simple trisaccharides are major components of the urinary free-glycans even in healthy individuals (Parkkinen and Finne 1983; Fu and Zopf 1999). However, other such glycans that are associated with cancer remain poorly characterized.

In this study, we have extensively investigated urinary free-glycans with the aim of identifying novel tumor marker candidates. Urine samples were obtained from patients with gastric cancer, pancreatic cancer and cholangiocarcinoma, as well as normal controls. There was a wide variety of glycan species in the urine. Here, we focused on the acidic fraction in line with previous research on free-glycans in cancer. To detect cancer-associated alterations of the major and minor structures, fluorescently labeled glycans were separated by multidimensional HPLC. We also performed structural profiling of free-glycans in the urine. Levels of the characteristic glycan were compared by selected reaction monitoring (SRM). In adopting this approach, we identified a number of urinary acidic free-glycans, including some novel structures, with elevated levels in cancer patients. The characteristic structures of these glycans were; sialyllactose-related small glycans, sialyl Lewis X, lactose- or LacNAc-core glycans with LacNAc-extension and modifications of α1,3/4-fucose and/or 6-sulfate, free-*N*-glycans containing sialylation or β1,6-branch of 6-sulfo Lewis X, novel NeuAcα2-3Galβ1-4(+/−Fucα1-3) Xylα1-3Glc.

## Results

### Preparation and separation of 2-aminopyridine-labeled acidic free-glycans from urine

**Fig. 1:**
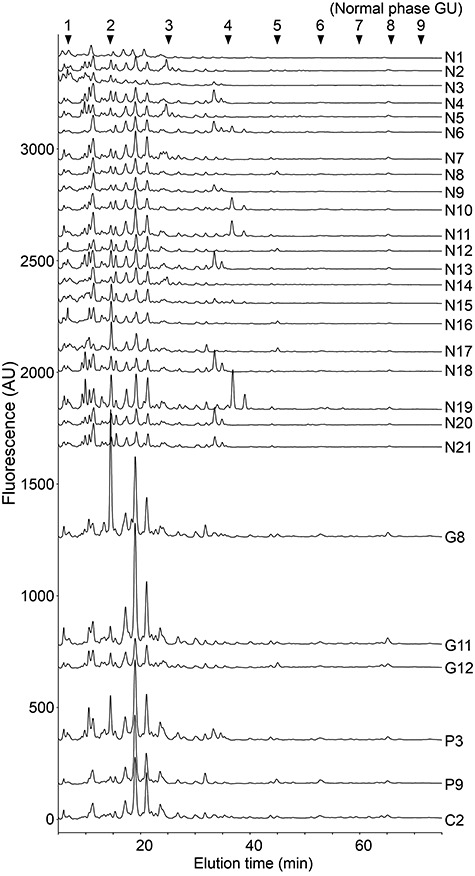
Normal phase chromatogram of PA-labeled free-glycans prepared from urine. Free-glycans in creatinine-adjusted urine from normal controls and cancer patients were labeled with PA and checked by normal phase HPLC. The amount of urine sample corresponded to 1 μg of creatinine. Chromatograms of 21 cases of normal controls (designated as N1–N21) together with representative cases of patients with gastric cancer (three cases; G8, G11 and G12), pancreatic cancer (two cases; P3 and P9) and cholangiocarcinoma (one case; C2) are shown.

**Fig. 2:**
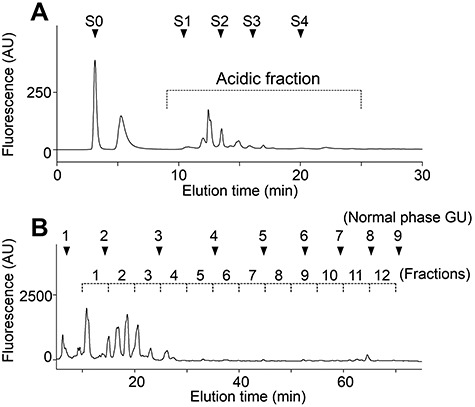
HPLC fractionation of urinary glycans. The PA-labeled glycans were sequentially fractionated by anion-exchange HPLC and normal phase HPLC. The representative chromatograms from a normal control (N8) are shown. (A) The elution profile of anion-exchange HPLC. PA-glycans were separated on a TSK-gel DEAE-5PW column and the acidic fraction was collected. Arrowheads S0–S4 indicate the elution positions of standard PA-*N*-glycans with 0–4 neuraminic acids (NeuAc). (B) The elution profile of normal phase HPLC. The acidic fraction from anion-exchange HPLC was fractionated in the range of GU1.4–8.7 on a TSK-gel Amide-80 column.

**Fig. 3:**
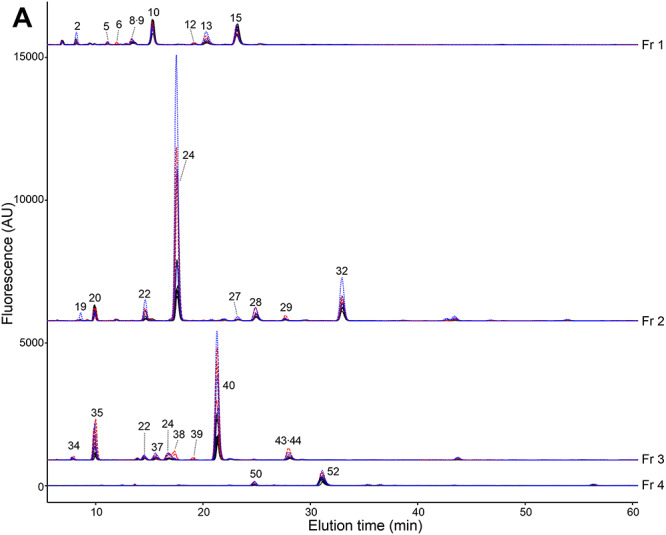

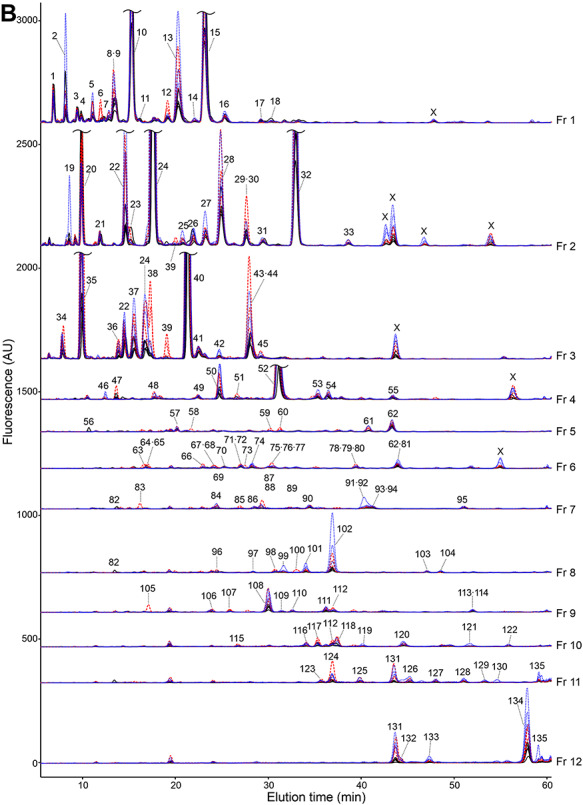
Reversed phase HPLC profiles of the urinary acidic free-glycans. PA-labeled free glycans fractionated by normal phase HPLC were further separated by reversed phase HPLC on a TSK-gel ODS-80Ts column. The amount of urine sample corresponded to 16 μg of creatinine. Representative overlaid chromatograms are shown from six normal controls (N5–N8, N11 and N14), black line; three gastric cancer patients (G8, G11 and G12), blue dotted line; two pancreatic cancer patients (P3 and P9) and one cholangiocarcinoma patient (C2), red dotted line. The major glycans comprising the fluorescent peaks are indicated as numbers. Estimated structures of the numbered glycans are shown in Table II in accordance with their basal structure and in supplementary data (Supplementary Table SII) in numerical order. X indicate peaks mainly composed of non-PA-glycan fluorescent materials. (A) The chromatograms of Fr 1–4, which contained more abundant glycans, are shown at a lower magnification of ×0.1. (B) The chromatograms of Fr 1–Fr 12.

Urine samples were obtained from 13 gastric cancer patients (G1–G13), 10 pancreatic cancer patients (P1– P10), 4 cholangiocarcinoma patients (C1–C4) and 21 normal controls (N1–N21; Table I and Supplementary Table SI). The urine samples were pretreated on a cation-exchange column and graphite carbon cartridge. Extracted free-glycans were then labeled with 2-aminopyridine (PA) at their reducing end. The NP-HPLC chromatograms of the total urinary PA-labeled free-glycans showed that the glycan amount was consistent among normal controls, but increased in some cancer patients (Figure 1). We reasoned that the urinary glycans were likely to exhibit too much structural diversity to perform a complete analysis of them all. In this study, we focused on acidic glycans because several acidic cancer-associated modifications of glycans were previously reported, including sialyl Lewis A/X and sulfation. The PA-glycans were separated by DEAE anion-exchange HPLC, and the acidic glycan fraction was collected and further separated into 12 fractions (Fr. 1–12) by NP-HPLC in the range of GU1.4–8.7 (Figure 2). Each fraction was then separated by RP-HPLC. Overlaid chromatograms from representative cases are shown in Figure 3.

**Table I:** Clinical information of the patients with gastric cancer, pancreatic cancer and cholangiocarcinoma

Case No.^a^	Sex	Age	CA19-9 (U/mL)^b^	CEA (ng/mL)^b^	ABO Blood type	Creatinine (mg/dL)
G1	F	61	4	1.1	B	32.1
G2	F	65	67	1.0	A	384.7
G3	M	65	206	60	B	115.3
G4	M	78	568	6.2	AB	160.6
G5	F	70	<2	1.2	B	319.4
G6	M	64	4	7.5	O	190.4
G7	M	72	76	1.7	A	115.7
G8	M	74	3	1.1	A	186.4
G9	M	74	7	4.4	AB	62.5
G10	M	59	1063	27.4	A	28.6
G11	F	67	2	1.2	O	162.9
G12	F	62	16	549.9	O	187.1
G13	M	60	1187	4.5	A	254.3
P1	M	48	3311	3.7	A	69.4
P2	F	58	>100,000	560.2	B	180.5
P3	M	68	>100,000	220.5	A	439.5
P4	F	50	16,421	11.5	A	50.5
P5	F	66	<2	3.0	B	159.5
P6	M	62	46,597	4.8	A	270.1
P7	M	72	20,124	13.1	A	51.5
P8	F	62	371	7.3	A	116.2
P9	M	64	>100,000	162.9	O	187.7
P10	M	77	>100,000	42.0	A	59.7
C1	F	55	29,046	206.0	O	74.6
C2	M	65	32,678	1673.1	AB	363.8
C3	F	78	>100,000	164.4	O	45.4
C4	M	74	81,803	156.2	O	153.4

^a^G, P and C indicate gastric cancer, pancreatic cancer and cholangiocarcinoma, respectively.

^b^Cutoff values of CA19-9 and CEA are 37 U/mL and 5 ng/mL, respectively.

We assigned PA-glycan structures based on elution positions of NP- and RP-HPLC (two-dimensional HPLC mapping) and mass spectrometry in combination with enzymatic digestions, acid treatments and periodate oxidation. Analysis of the representative structures is described in the following sections. In all, 135 PA-glycans were identified in this study that comprised mixtures of various core structures. We categorized these glycans into seven groups as follows; lactose-core glycans (35 structures), *N*-acetyllactosamine (LacNAc) type-II-core glycans (25 structures), free-*N*-glycans (25 structures), free-mucin-type glycans (12 structures), Xyl-Glc-core glycans (2 structures), other sialylated glycans (9 structures) and other hexuronylated glycans (19 structures). The glycans are summarized in Table II in accordance with their basal structure and in supplementary data (Supplementary Table SII) in numerical order. The glycans are depicted according to the “Symbol Nomenclature for Glycans” (Varki et al. 2015a). Moreover, elevated glycan levels in cancer patients were evident from inspection of the RP-HPLC chromatograms (Figure 3). SRM assays were performed for quantitative comparison as described later.

### Structural analyses of lactose-core glycans

**Fig. 4:**
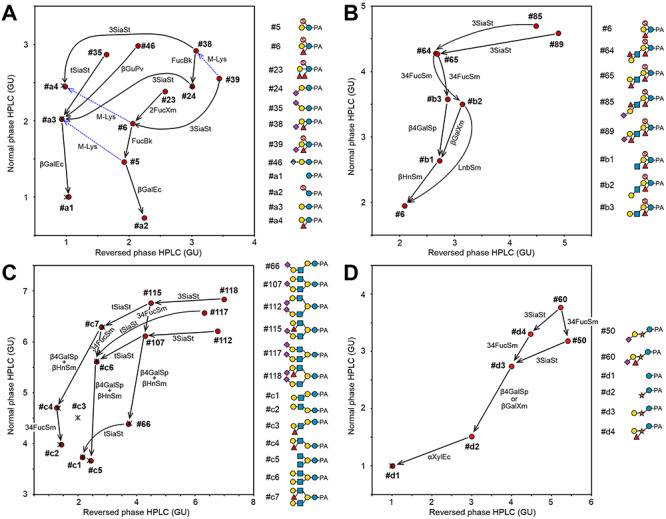
Structural analysis of PA-glycans by two-dimensional HPLC mapping. The elution positions of PA-glycans on HPLCs of normal phase and reversed phase were standardized into glucose units (GU). Red circles and asterisks indicate the elution positions of the sample glycans and the standard glycans, respectively. The structures designated as #a1–a4, b1–b4, c1–c7 and d1–d4 are the digested glycans and standard glycans, which were not detected as their forms in the analysis. Solid arrows indicate shifts of the elution positions of the glycans by glycosidases. Dotted arrows with “M-Lys” indicate shifts by methanolysis. The glycosidases used were as follows: 3SiaSt, α-neuraminidase under the conditions for nonreducing terminal α2,3-linkages (from *S. typhimurium*); tSiaSt, α-neuraminidase for nonreducing terminal α2,3/6-linkages (from *S. typhimurium*); βGuPv, β-glucuronidase (from *P. vulgata*); βGalEc, β-galactosidase for PA-disaccharides (from *E. coli*); βGalXm, β-galactosidase with specificity for β1,3 > 6 > 4 (from *X*. *manihotis*); βGalSp, β1,4-galactosidase (from *S. pneumoniae*); FucBk, α-fucosidase (from bovine kidney); 2FucXm, α1,2-fucosidase (from *X*. *manihotis*); 34FucSm, α1,3/4-fucosidase (from *Streptomyces* sp. 142); bHnSm, β-1,3/4/6-*N*-acetylhexosaminidase (from *S. plicatus*); LnbSm, lacto-*N*-biosidase (from *Streptomyces* sp. 142); and aXylEc, α-xylosidase (from *E. coli*). (A) Representative small lactose-core glycans. (B) Representative LacNAc extended lactose-core glycans containing Lewis A and Lewis X. (C) Lactose-core glycans with branches of LacNAc-extensions (lacto-*N*-neohexaose backbone). (D) The Xyl-Glc-core glycan #50 and #60. The digestions suggested partial structural information of NeuAcα2-3Galβ1-Xylα1-Glc-PA with/without α3/4-fucosylation on the xylose residue.

**Fig. 5:**
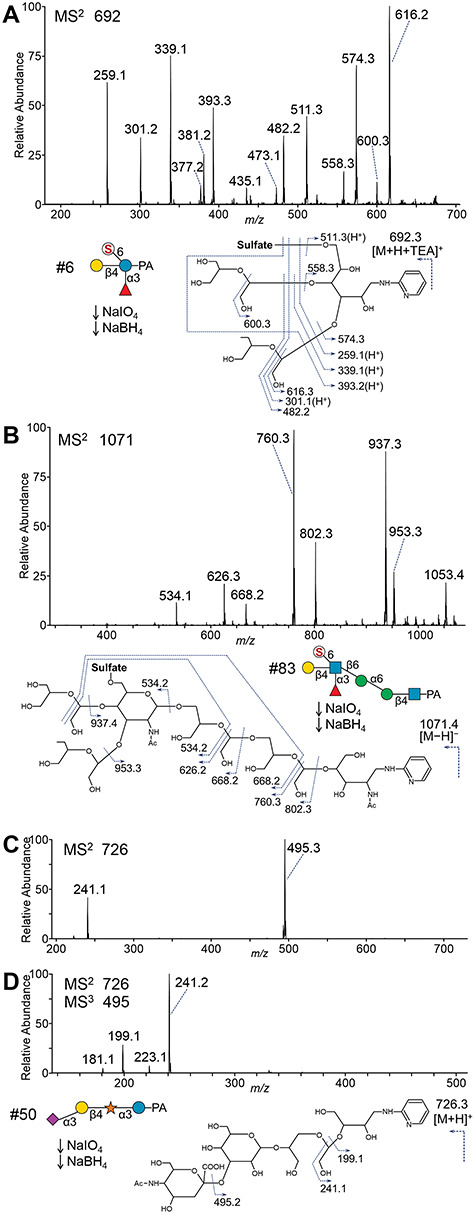
Structural analysis of PA-glycans by MS^n^ after periodate cleavage. PA-glycans were oxidatively cleaved with sodium periodate and reduced with sodium borohydride. The products were analyzed by MS^n^. (A) Positive mode MS^2^ spectrum of the cleaved product of PA-glycan #6 from the protonated ion with triethylamine at *m/z* 692. (B) Negative mode MS^2^ spectrum of the product of sulfated and fucosylated free-*N*-glycan #83 from the de-protonated ion at *m/z* 1171, suggesting 6-sulfo-Lewis X linked to α1,6-Man-arm by β1,6-branching. (C and D) MS^n^ analysis of the Xyl-Glc-core glycan #50, revealing the partial sequence information of NeuAc2-3Hex1-4Pen1-3Hex-PA. (C) Positive mode MS^2^ spectrum of the product of #50 from the protonated ion at *m/z* 726. (D) MS^3^ spectrum of the B-ion composed of C_2_H_2_O-tetrose-PA at *m/z* 241 from MS^2^ of #50.

Glycans containing lactose (Galβ1-4Glc) as the core structure, which resemble milk oligosaccharides, accounted for the majority of the acidic fraction of urinary free-glycans. Some of the cancer patients showed increased levels of lactose-core glycans, which were mainly composed of relatively small, sialyl and/or sulfated lactose. Two-dimensional HPLC mapping analysis of representative small lactose-core glycans is shown in Figure 4A. In all cases, PA-glycan #24, 3′-sialyl lactose was found to be the most abundant. PA-glycan #35, 6′-sialyl lactose was also present at relatively high levels. A trisaccharide #22, which has a reducing terminal Man, was estimated to be NeuAcα2-3Galβ1-4Man-PA, 3′-sialyl-epilactose, an epimerized form of 3′-sialyl lactose (#24) at the C-2 position. Because of poor separation in the two-dimensional mapping, the reducing terminal Hex-PA residues were also analyzed by an HPLC for monosaccharides (Supplementary Figure S1). Similarly, the corresponding C-2 epimers were also detected in other structures as minor components. A fucosylated glycan #38 corresponding to 3′-sialyl-3-fucosyl lactose was digested into glycan #24 by bovine kidney fucosidase, but into 3-fucosyl Lac by *Salmonella typhimurium* α2,3-neuraminidase treatment. A sulfated glycan #39 (Hex_2_dHex_1_NeuAc_1_Sulfate_1_-PA) produced glycan #38 by methanolysis or a sulfated asialo-glycan #6 by *S. typhimurium* α2,3-neuraminidase. In addition, glycan #6 was digested into a sulfated lactose #5 by bovine kidney fucosidase. To determine the sulfate position, periodate oxidation analysis of glycan #6 was performed. The product was detected as an ion at *m/z* 692 [M + H + TEA]^+^ (Hex_2_dHex_1_Sulfate_1_-PA +2 × 2H −2 × CH_2_O), and the MS^2^ spectrum of the ion contained ions at *m/z* 259 and 339 corresponding to protonated forms of intact Hex-PA and sulfate-Hex-PA, respectively (Figure 5A). These findings were consistent with C-6 sulfation of the reducing terminal Glc. In summary, our results indicated that glycans #5, #6 and #39 were 6-sulfo- lactose and its modified structures. Also, PA-glycan #23 shifted into glycan #6 by *Xanthomonas manihotis* α1,2-fucosidase, and was assigned as 6-sulfo-2′,3-difucosyllactose. PA-glycan #46 corresponded to β-glucuronyllactose, which was digested into lactose by *Patella vulgata* β-glucuronidase.

A minor glycan #68 composed of Hex_3_NeuAc_2_-PA was deduced to be 3″,6′-disialyl-3′-β-galactosyllactose (Supplementary Figure S2). The galactosyllactose backbone is a structure commonly found in milk oligosaccharides (Sumiyoshi et al. 2004). PA-glycans #47 and #51 were, respectively, digested by coffee bean α-galactosidase and *Elizabethkingia meningoseptica* α-*N*-acetylgalactosaminidase and converged into glycan #23. The α-Gal/GalNAc moieties were attached to the lactose-core via a 1,3/4-linkage, which was confirmed by periodate cleavage. In addition, glycan #47 was detected in only some blood group B/AB cases of normal and cancer patients. Moreover, glycan #51 was only found in some blood group A/AB cases. Based on these results, we concluded that glycan #47 and glycan #51 were likely to contain blood group B- and A-antigens, respectively, and were consistent with sulfated forms of previously reported blood group-related free-pentasaccharides in urine (data not shown) (Björndal and Lundblad 1970; Lundblad and Svensson 1973b). PA-glycan #48 corresponded to GM2-tetrasaccharide, a 3′-sialyllactose with an additional β1,4-GalNAc. Glycan #11 was estimated to be a lactose with 2-fucosylation and sulfation at the Gal residue. The de-fucosylated form corresponded to glycan #2 (Supplementary Figure S3). The sulfate group was deduced to be on the C-4 or 6 position of Gal, although most likely on C-6 considering its similarity to the LacNAc-core glycan #17.

The lactose-core glycans with a LacNAc type-II-extension (i.e., Lacto-*N*-neotetraose backbone, Galβ1-4GlcNAcβ1-3Galβ1-4Glc-PA) were found to be relatively minor components. Their lactose-core structures were also frequently modified with 3-fucosylation and/or 6-sulfation. Modifications of the antennal LacNAc, α2-3/6-sialylation, 3-fucosylation (Lewis X) and/or 6-sulfation of the GlcNAc were also observed. The sialyl Lewis X extension was assigned by sequential digestion with *S*. *typhimurium* α2,3-neuraminidase, *Streptomyces* sp. α1,3/4-fucosidase, *Streptococcus pneumoniae* β1,4-galactosidase and *Streptomyces plicatus* β-*N*-acetylhexosaminidase (Figure 4B). In addition, 6-sulfation of LacNAc was confirmed by sequential digestion with jack bean β-galactosidase and human placenta β-*N*-acetylhexosaminidase after removal of NeuAc from the MS^2^ spectrum following periodate cleavage (Supplementary Figure S4) (Hepbildikler et al. 2002; Murakami et al. 2007). Glycans #59, #66, #71 and #75 possessed 6′-sialyl-LacNAc extensions. PA-glycans #70, #72, #84, #89 and #96 possessed 3′-sialylated type-II LacNAc extensions, and those of #72, #84, #89 and #96 were sialyl Lewis X. Among these glycans, #59, #72 and #75 also comprised C-6 sulfation on the antennal GlcNAc. Glycans #58, #63 and #65 had sulfated asialo-glycans with antennal Lewis X. The glycan #58 contained two sulfate groups at the C-6-positions of the reducing end Glc and the antennal GlcNAc residues. Sialylated glycans containing a branched type of Lacto-*N*-neohexaose (Galβ1-4GlcNAcβ1-6(Galβ1-4GlcNAcβ1-3) Galβ1-4Glc) were also detected (#107, #112, #115, #117 and #118). Among them, #115 and #118 contained Lewis X on the β1,6-arm (Figure 4C). Although most of extensions of the lactose-core were type-II LacNAc, glycans with LacNAc type-I (i.e., Lacto-*N*-tetraose backbone, Galβ1-3GlcNAcβ1-3Galβ1-4Glc) were also found as Lewis A and sialyl Lewis A structures (#64 and #85; Figure 4B).

### Structural analyses of type-II N-acetyllactosamine-core free-glycans

Glycans containing LacNAc type-II-core were also major components of the urinary acidic free-glycans. The structural features of LacNAc-core glycans resemble those of lactose-core glycans (Table II, LacNAc-core). Among this group, 6′-sialyl LacNAc (#40) were most abundant, followed by 3′-sialyl LacNAc (#32). PA-glycan #43 corresponded to sialyl Lewis X tetrasaccharide, and #30 corresponded to its 6-sulfated form. Glycans #12 and #16 were asialo-sulfo-Lewis X with the former sulfated at C-6 of GlcNAc and the latter sulfated at C-3 of Gal. PA-glycan #29 was the 6-sulfated form of 6′-sialyl LacNAc. PA-glycan #80 was similar to the lactose-core #68, and proposed to be NeuAcα2-6(NeuAcα2-3Galβ1-3) Galβ1-4GlcNAc. PA-glycan #17 was similar to the lactose-core of #11, and deduced to be 6′-sulfo-2′-fucosyl LacNAc (Supplementary Figure S3).

The LacNAc-core with additional LacNAc structures were also detected. The simple α2,3- and α2,6-sialylated type-II di-LacNAc corresponded to #62 and #78, respectively. Glycans containing a Lewis X-core were extended with the following: 6-sulfo-Lewis X (#67), 6′-sialyl-6-sulfo-type-II LacNAc (#79), 3′-sialyl -type-II LacNAc (#90), sialyl Lewis X (#98) and sialyl Lewis A (#100). In the case of glycan #100, the backbone was a di-LacNAc but with a type-I → type-II hybrid structure. Glycan #104 was related to glycan #68, and assigned as NeuAcα2-3Galβ1-4GlcNAcβ-(NeuAcα2-3Galβ1-3) Galβ1-4GlcNAc-PA. PA-glycan #103 corresponded to linear tri-LacNAc type-II with α2,3-sialylation. PA-glycan #113 and #114 were detected as overlapping peaks by RP-HPLC (Figure 3B, Line Fr9), and then subsequently separated by a second NP-HPLC. These glycans contain a branched tri-LacNAc type-II backbone, similar to branched lacto-*N*-neohexaose, with two α2,3-sialylations (#113) or one α2,3- and one α2,6-sialylation (#114).

### Structural analyses of free-N-glycans

Free-*N*-glycans were found to be major components of late-eluted fractions from the NP-HPLC (Fr 7–12). Most of these glycans had typical sialylated mono- and bi-antennary structures. The sialylations were mainly α2,6-linkages. Two types of reducing end structures, GlcNAc_1_-type and GlcNAc_2_-type, were found. Intriguingly, some cancer patients showed elevated levels of these *N*-glycans. Among the GlcNAc_1_-type glycans, major components were α3-Man-arm-monoantennary structures #102 and #108 and biantennary structures #124 and #131, of which the antennae were type-II LacNAc with or without α2,6-sialylation (Figure 3A, Table II free *N*-glycans (GlcNAc_1_)). Sialyl monoantennary glycans containing α6-Man-arm were detected as minor components (#101 and #110). PA-glycan #91 was similar to #102, but the antenna was 6′-sialyl LacdiNAc. These monoantennary structures were identical to those previously found in tumor tissue analysis (Yabu et al. 2013b). PA-glycans #119 and #130 were assumed to be mono- and bi-antennary glycans containing

**Table II:** Estimated structures of acidic free-glycans from urine

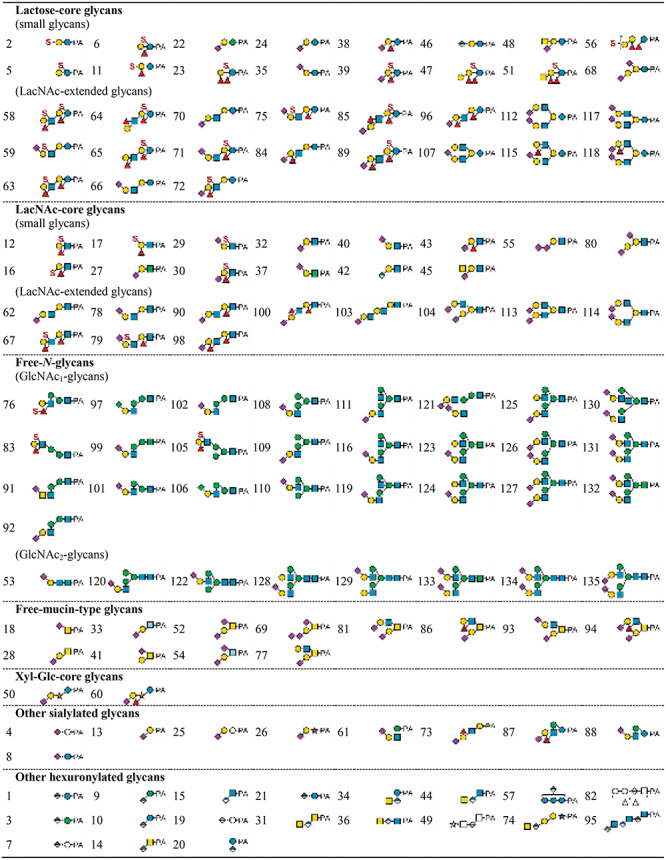

Glycan numbers are from Figure 3. Monosaccharide symbols are according to the symbol nomenclature for glycans, and indicated as follows: blue circle, Glc; blue square, GlcNAc; green circle, Man; green square, ManNAc; yellow circle, Gal; yellow square, GalNAc; light-blue square, TalNAc; red triangle, Fuc; purple diamond, Sialic acid (NeuAc); green diamond, KDN; divided diamond with blue upper side and white lower side, GlcA; white circle, unassigned Hex; white square, unassigned HexNAc; white triangle, unassigned dHex; divided diamond, unassigned HexA; S, sulfate.

 bisecting GlcNAc. PA-glycan #121 was concluded to be a biantennary Man_2_GlcNAc_1_-core glycan containing an α3-Man-arm with two sialyl LacNAc antennae attached by β1,2- and β1,4-linkages, respectively. Minor components, #97 and #106, were concluded to be monoantennary Man_2_GlcNAc_1_-PA glycans with α2,6-linked KDN (Yabu et al. 2013a).

We also identified unusual sulfated free-*N*-glycans. Glycan #83 (Figure 3B, Fr 7) was composed of Hex_3_HexNAc_2_dHex_1_Sulfate_1_-PA at *m/z* 1316 [M + H + TEA]^+^. The presence of an antenna of 6-sulfo-Lewis X was confirmed by glycosidase digestions (data not shown). To obtain more detailed linkage information, periodate oxidation was performed and the product was detected with *m/z* 1071 [M − H]^−^ (Hex_3_HexNAc_2_dHex_1_Sulfate_1_-PA +3 × 2H, −5 × CH_2_O) in negative-mode MS (Figure 5B). In the MS^2^ spectrum, ions at *m/z* 937 and 953 were detected corresponding to losses of nonreducing terminal unmodified Hex and dHex residues, respectively. In addition, an ion of antennal structure *m/z* 534, indicated C-6 sulfation of the antennal GlcNAc. Furthermore, ions at *m/z* 760 and 626 were consistent with products of cleavage at the β4-Man residue and at the α6-Man residue with the antenna at the C-6 position, respectively. These results indicated that the antenna of the glycan #83 was attached to α6-Man-arm via a β1,6-linkage. The structure of glycan #105 (Figure 3B, Fr 9) corresponded to glycan #83 with an addition of a α1,3-Man residue to the core. Glycan #76 (Figure 3B, Fr 6) was also composed of Hex_3_HexNAc_2_dHex_1_Sulfate_1_-PA at *m/z* 1316 [M + H + TEA]^+^, and expected to be a mono-antennary structure with sulfated Lewis X. This glycan showed resistance against fucosidases until methanolysis. After de-sulfation, the glycan was digested by α3/4-fucosidase resulting in an α6-Man-arm structure with one type-II LacNAc antenna (data not shown). The sulfate group position was confirmed by mass spectrometry after periodate oxidation as shown in Supplementary Figure S5. These results indicated that glycan #76 possess a unique Lewis X antenna containing a 4-sulfo-Fuc residue.

Free-*N*-glycans remaining in their GlcNAc_2_-core structure were mostly typical sialylated mono- or bi-antennary structures. Among them, the di-α2,6-sialyl bi-antennary glycan #134 was the most abundant (Figure 3B, Line Fr 12). An exception was glycan #53 assigned as NeuAcα2-6Galβ1-4GlcNAcβ1-4GlcNAc-PA by glycosidase digestions and periodate oxidation (Supplementary Figure S6), which was detected in both cancer patients and normal controls (Figure 3B, Line Fr 4). This tetrasaccharide was previously found on serum transferrin from patients with Congenital Disorders of Glycosylation (Bengtson et al. 2016; Zhang et al. 2016).

### Structural analyses of free-mucin-type glycans

The free form of mucin type *O*-glycans were also observed, although analyses of the peaks from HPLC did not show any significant cancer-related changes. Glycan #18 was matched with sialyl Tn. The core 1-type glycans comprised simple mono- to tri-sialyl structures (#28, #41, #52 and #69). The core 2-type glycans were typical mono- or di-sialylated forms (#77, #81 and #93) in addition to a α1,3-fucosylated structure at β1,6-GlcNAc (#86 and #94). Glycans #33 and #54 contained a reducing terminal *N*-acetyltalosamine (TalNAc), corresponding to the C-2-epimer of GalNAc. Thus, glycans #33 and #54 match the epimerized forms of the core 1-type glycans #28 and #52, respectively (Figure 3B, Line Fr2 and Fr4).

### Structural analysis of novel Xylα1-3Glc-core glycans

We detected two glycans containing an internal Pen residue (#50 and 60), and suspected that they contained Xylα1-3Glc-core known as components of neutral urinary free-glycans (Lundblad and Svensson 1973a; Toyota et al. 1994). Glycan #50, which comprises Hex_2_Pen_1_NeuAc_1_-PA at *m/z* 844 [M + H]^+^, was observed in all cases (Figure 3, line Fr4). The sialic acid was removed by *S. typhimurium* neuraminidase under α2,3-specific conditions. One Hex-residue was then slowly removed by β-galactosidases from either *S. pneumoniae* or *X. manihotis*, indicating a β-linkage with an ambiguous position of Gal. The residual disaccharide was digested by *Escherichia coli* α-xylosidase yielding a reducing terminal Glc (Figure 4D; Supplementary Figure S1). To confirm linkage positions, mass spectrometric analysis after periodate oxidation was performed. The glycan was detected as the protonated ion at *m/z* 726 [M + H]^+^ (Hex_2_Pen_1_NeuAc_1_-PA +2H, −4CH_2_O). The base peak ion of the MS^2^ spectrum at *m/z* 495 corresponded to the de-sialylated Y-ion, and was subjected to further fragmentation (Figure 5C). In the MS^3^ spectrum, an ion at *m/z* 199 was detected corresponding to PA-labeled tetrose, which suggested glycosidic occupation of the C-3 hydroxyl group of the reducing end Hex-residue. In addition, an ion at m/z 241 was detected, which corresponded to the PA-tetrose with additional C_2_-fragment (42 Da), indicating periodate-cleavage between C-2 and C-3 with free hydroxyl groups of the internal Pen-residue (Figure 5D). These results indicated that the glycan #50 was a novel Xyl-Glc-core glycan, NeuAcα2-3Galβ1-4Xylα1-3Glc-PA. A fucosylated glycan #60 was found in only some pancreatic cancer and cholangiocarcinoma patients as a fluorescent peak (Figure 3B, Fr 5). This glycan was digested by α3/4-fucosidase resulting in glycan #50. In addition, the pentose-residue showed resistance to periodate cleavage (data not shown). From these results, glycan #60 was deduced to be a novel fucosyl xylose containing glycan, NeuAcα2-3Galβ1-4(Fucα1-3) Xylα1-3Glc-PA.

### Structural analyses of other sialylated glycans

Urine also contained sialylated glycans with alternative backbone structures. Sialyl Hex disaccharides, #4, #8 and #13 were detected in Fr. 1 (Figure 3 and Supplementary Figure S1). Glycan #25 and #26 were trisaccharides containing NeuAcα2-3Galβ4-sequence, similar to glycan #24, 3′-sialyllactose (Figure 3, line Fr2). After digestion with α2,3-neuramindiase (*S*. *typhimurium*) and β-galactosidase (*E. coli*), the reducing end of glycan #25 was a Hex-PA. However, the position on the 2D-HPLC map did not correspond with Glc, Man, Gal or Tal (data not shown). The reducing end of glycan #26 was consistent with Xyl. The glycan NeuAcα2-6Galβ1-4GlcNAcβ1-2Man-PA (#61) corresponded to a free-form of Core M1 (GlcNAcβ1-2Man)-type *O*-mannosyl glycan. PA-glycan #73 was a sialyl Lewis A attached via C-3 of Gal, which corresponded to a peeling product of mucin type glycans previously identified in serum *O*-glycan analysis (Tanaka-Okamoto et al. 2016). We also found two glycans #87 and #88 containing an Hex_3_HexNAc backbone, which were deduced not to have a lactose-core. Instead, these glycans comprised an unusual Galβ1-4GlcNAcβ1-2Manα1-6Glc-PA resembling *N*-glycans and Core M1-*O*-glcyans. Moreover, our analyses showed that glycans #87 and #88 possessed sialyl Lewis X-modification and α2,6-sialylation, respectively (Supplementary Figure S1 and Supplementary Figure S7).

### Structural analyses of other hexuronylated glycans

Hexuronic acids (HexA)-containing glycans were also noticeable components in our analyses (Table II, other hexuronylated glycans). These glycans were stably detected in samples from both cancer patients and healthy controls. Several hexuronylated Hex or HexNAc disaccharides were major components of the earlier NP-HPLC fractions, Fr. 1 and 2 (Figure 3 and Supplementary Figure S1). Among them, β3-glucuronyl Glc and GlcNAc (#10 and #15) were most abundant and the latter was corresponded to a disaccharide unit of hyaluronan (Figure 3A, low magnification). In addition, urine showed a variety of HexA-containing structures, including a Pen-capped structure, a glucuronyl maltotriose, a free-form of a protein-linkage structure of glycosaminoglycan, a B-blood group related structure and hyaluronan hexasaccharide (#49, #57, #74, #82 and #95). We noticed that some glycans possessed an additional nonreducing terminal HexNAc attached to the sub-terminal GlcA (#31, #34, #36, #44 and #74). The HexNAc residue was revealed to be an α1,4-linked GalNAc by treatment with α-*N*-acetylgalactosaminidase and periodate oxidation (Supplementary Figure S8). This additional GalNAc was similar to the capping structure of glycosaminoglycans previously reported from in vitro experiments (Manzi et al. 1995; Miura and Freeze 1998; Kitagawa et al. 1999).

### Comparison of acidic free-glycan levels by SRM

HPLC-based free-glycan profiling of urine from cancer patients and normal controls suggested some of these glycans may be present at elevated levels in association with cancer. Fluorescent detection in HPLC is convenient and has the advantage that the fluorescence intensity is equivalent among structurally different glycans (Natsuka and Hase 1998). However, quantification is often difficult because of lower sensitivity compared with MS detection. In addition, peak fusion and overlap due to the presence of many glycan species in the sample can be problematic. Therefore, in order to compare each glycan level from individuals, we also performed SRM. For these measurements, we selected glycans that were found to be elevated in cancer patients by HPLC analysis, as well as some of the major glycans that displayed little change between cancer patients and normal controls. The ionization conditions, including Q3 value and collision energy, were set for each individual glycan to facilitate efficient detection. To enhance detection, we used eluates containing triethylammonium acetate. In terms of the detection of PA-glycans in positive ion mode, formation of a triethylamine additive improved sensitivity and suppressed degradation of sulfate modifications. The addition of triethylamine resulted in monovalent ions of PA-glycans, which limited the scan range of the mass spectrometer used for SRM, so that glycans with an *m/z* lower than 2000 were included in the measurements. Although the number of samples used in the present study is limited and insufficient for statistical analysis, the SRM semiquantitative measurements showed interesting differences in the levels of glycans, depending on backbone structure and modifications.

**Fig. 6:**
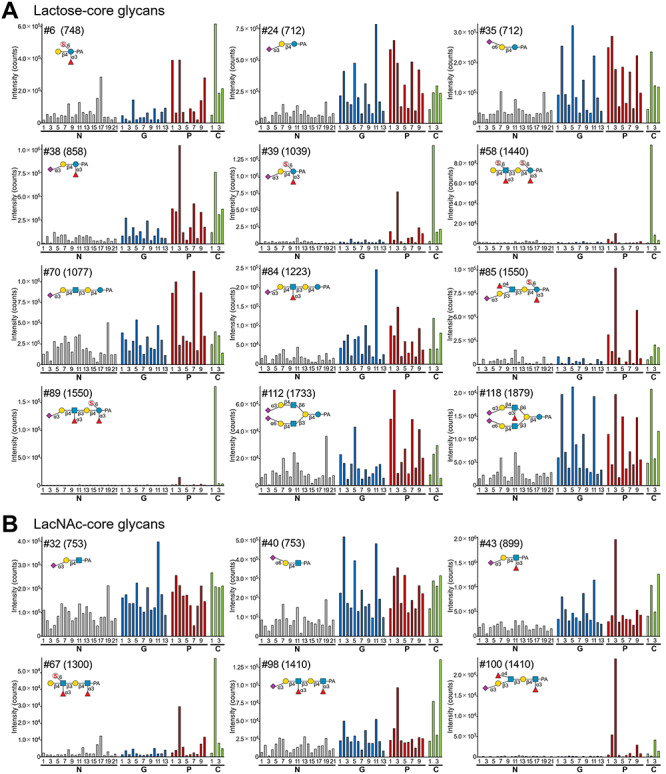

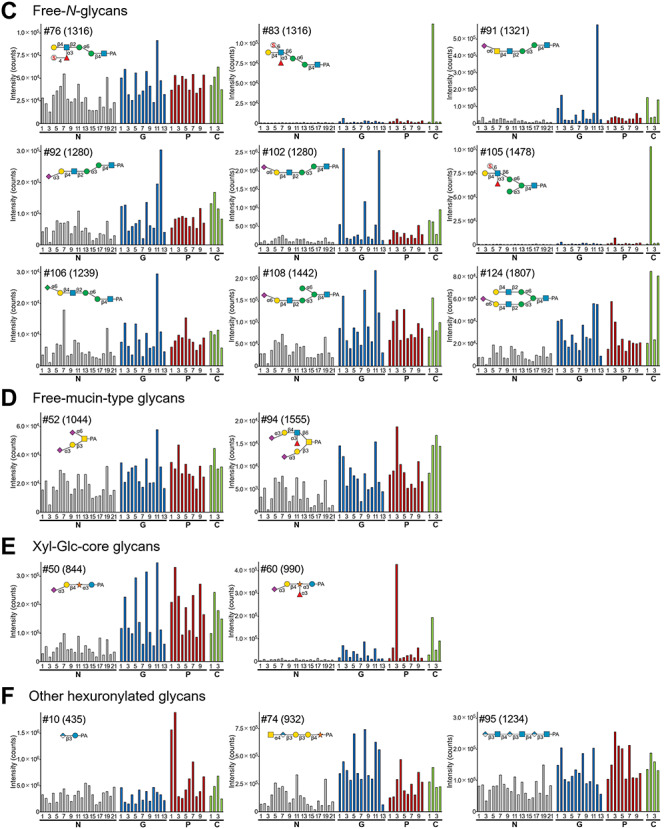
Levels of representative free-glycans in the urine from SRM analysis. The amount of urine sample corresponded to 8 μg of creatinine, but exceptionally 0.8 μg of creatinine for glycans #10, #24, #32, #34, #40, #50 and #52. The peak areas in the extracted ion chromatogram (XIC) of SRM measurements are shown. The levels of the glycans are indicated by bars as follows: normal controls (N1–21), gray; gastric cancer patients (G1–13), blue; pancreatic cancer patients (P1–10), red; and cholangiocarcinoma patients (C1–4), light green. In each glycan panel, glycan number, estimated structure and the mass values of Q1 are indicated. Also, the values of Q1 and Q3 are shown in Supplementary Table SIII. (A) Lactose-core glycans #6, #24, #35, #38, #39, #58, #70, #84, #85, #89, #112 and #118 are shown. (B) LacNAc-core glycans #32, #40, #43, #67, #98 and #100 are shown. (C) Free-*N*-glycans #76, #83, #91, #92, #102, #105, #106, #108 and #124 are shown. (D) Mucin-type free-glycans #52 and #94 are shown. (E) Xyl-Glc-core glycans #50 and #60 are shown. (F) Other uronylated glycans #10, #74 and #95 are shown.

 The results of SRM measurements of the glycans are shown in Figure 6 and Supplementary Figure S9. The *p*-value of each glycan by Mann–Whitney *U* tests and fold change of the mean values in each cancer patient group compared with normal controls are shown in Supplementary Table SIV. For clarity, a summary of the results pertaining to each glycan group are considered in turn.

 Among the lactose-core glycans, most of these compoundsdisplayed elevated levels in cancer patients (  Figure 6A ; Supplementary Figure S9 A). Backbone structures and modifications of glycans seemed to influence the cancer-related increase in the level of these compounds. The simple sialyllactoses (#24 and #35) showed elevated levels in cancer patients (Figure 6A). Elevation of 3′-sialyllactose (#24) in cancer patients was consistent with previous reports (Shimada et al. 1995; Zhang et al. 2013). The 3-fucosyllactoses with 3′-sialylation and/or 6-sulfation (#6, #38 and #39) were present at elevated levels in some pancreatic cancer and cholangiocarcinoma patients, espec-ially cases P3 and C2 (Figure 6A). However, lactose with sulfation and 2′-fucosylation on the Gal residue (#11 in Supplementary Figure S9A) did not show any apparent increase. The levels of lactose-core glycans with LacNAc-extensions showed a slightly different trend to those of the small lactose-core glycans. Sialylated lacto-*N*-neotetraoses and β1,6-branched lacto-*N*-neohexaoses displayed less marked levels of elevation (#70 and #112 in Figure 6A; #66 and #117 in Supplementary Figure S9A). By contrast, glycans containing sialylation and Lewis X showed a pronounced increase in cancer patients (#84 and #118 in Figure 6A; #96 and #115 in Supplementary Figure S9A). In only one case of a cholangiocarcinoma patient C2, greatly increased levels of glycans with 6-sulfation on the reduc-ing terminal Glc and/or the antennal GlcNAc were observed (#58 and #89 in Figure 6A; #59, and #75 in Supplementary Figure S9A). The level of #89 showed a slight increase in some other pancreatic cancer and cholangiocarcinoma patients. The levels of a glycan with sialyl Lewis A #85 increased in two pancreatic cancer patients P3 and P9 (Figure 6A).

 LacNAc-core glycans are structurally similar to lactose-core glycans but showed a reduced cancer-related elevation (  Figure 6B , Supplementary Figure S9B). The level of 3′-sialyl LacNAc (#32) compared to that of 3′-sialyllactose (#24) is one such example. The small glycans of 6′-sialyl LacNAc (#40) and sialyl Lewis X tetrasaccharide (#43) showed increased levels in cancer patients. Lewis X-core glycans with extension of 6-sulfo-Lewis X and sialyl Lewis X (#67 and #98) displayed increased levels in some pancreatic cancer and cholangiocarcinoma patients. The only LacNAc core glycan showing relatively high cancer-specific elevation comprised Lewis X-core with antennal sialyl Lewis A (#100). This glycan showed increased levels in some pancreatic cancer and cholangiocarcinoma patients compared with normal controls and gastric cancer patients.

 Among free-*N*-glycans, several sialylated or sulfated structures showed a tendency to increase in cancer patients (  Figure 6C , Supplementary Figure S9C). The level of glycans containing α2,6-sialylated antenna and Man_2_GlcNAc_1_- or bisecting GlcNAc-Man_3_GlcNAc_1_-core (#91 and #102 in Figure 6C; #119 and #121 in Supplementary Figure S9C) increased in cancer patients, particularly in some gastric cancer patients (G2 and G12). The monosialylated biantennary glycans, such as #124, showed elevated levels in some cancer patients. The glycans containing 6-sulfo-Lewis X antenna attached via a β1,6-linkage to the α1,6-Man residue (#83 and #105 in Figure 6C) were also increased. In particular, patient C2 showed a marked elevation, similar to Lactose- and LacNAc-core glycans with 6-sulfation. By contrast, less dramatic increases in the levels of α2,6-sialylated monoantennary Man_3_GlcNAc_1_- or Man_3_GlcNAc_2_-core glycans (#108 in Figure 6C; #120 in Supplementary Figure S9C) and the α2,3-sialylated mono-antennary Man_2_GlcNAc_1_-core glycan (#92 in Figure 6C) were observed. No apparent increase was observed for the KDN containing glycan (#106) and the unique Lewis X-antennary glycan containing 4-sulfo-Fuc (#76). SRM measurements in this study were targeted at PA-glycans with a monovalent ion of less than *m/z* 2000, but some free-*N*-glycans exceeded this value. Typical di-α2,6-sialyl biantennary glycans #131 and #134 were major components found in all samples. Given the high purity of their fluorescence peaks, we compared the glycan levels of #131 and #134 based on their fluorescent signal (Supplementary Figure S10). The Man_3_GlcNAc_1_-core glycan (#131) was elevated in gastric cancer and cholangiocar-cinoma patients. The Man_3_GlcNAc_2_-core glycan (#134) was also elevated in these patients but to a lesser extent.

 SRM measurements were performed for representative structures of the free-mucin-type glycans (  Figure 6D , Supplementary Figure S9D) and the group of other hexuronylated glycans (Figure 6F and Supplementary Figure S9F). The levels of these glycans was elevated, but to a lesser extent in cancer patients.

 The levels of two sialylated Xyl-Glc-core glycans identified in this study (#50 and #60) were both increased in cancer patients ( Figure 6E ). The glycan #50 showed elevated levels in cancer patients, although it was relatively abundant in normal controls. Furthermore, glycan #60, in which Xyl is α3-fucosylated, showed low levels in normal controls and increased levels in several gastric cancer, pancreatic cancer and cholangiocarcinoma patients, especially P3 and C2. 

 For the group of other sialylated glycans, analyzed structures showed a tendency to increase in cancer patients (#13, #61, #73 and #88 in Supplementary Figure S9E). Among them, elevated levels of sialyl Lewis A-Gal #73 were found in some pancreatic cancer patients.

### Principal component analysis

 In an attempt to visualize the relationship between structural featuresof glycans and cancer patients, principal component analysis (PCA), an unsupervised method, was performed. Data of the levels of 68 glycans from SRM or fluorescence detection (Supplementary Figure S11 ) (  Holst et al. 2016 , 2019) were used. In score plots of PC1-PC2 and PC1-PC3, the distributions of cancer patients and normal controls were partially segregated (Supplementary Figure S11A and C). Due to the presence of individuals with cancer who did not show apparent changes in glycan levels (e.g. G7 and P8), complete group separation could not be achieved. The PC1 axis (48.6%) appeared to reflect overall changes in glycan levels. The PC2 axis (13.8%) seemed to reflect modifications on the glycans (Supplementary Figure S11A and B). For most glycans, locational proximity on the PC1-PC2 loading plot showed association with similarity of patterns and changes in glycan levels (Figure 6, S9 and S10). However, this association was apparent even though elevations of several glycans in cancer patients were to a lesser extent. In particular, most 6-sulfated glycans (e.g. #39, #67, #89 and #105) were located in the bottom area of the plot regardless of their core-structures, which was related to their extreme elevation in cancer patient C2. Several glycans with 3-fucosylation (e.g. #38 and #60) and glycans with sialyl Lewis A (#73, #85 and #100) were located in the lower area of the loading plot. This clustering was related to the distribution of some pancreatic cancer and cholangiocarcinoma patients on the PC1-PC2 score plot, in accordance with their increase observed for these patients, especially P3. The PC3 axis (8.3%) appeared to reflect the core structures in addition to the modifications of the glycans, referring to the PC1-PC3 loading plot (Supplementary Figure S11D). Free-*N*-glycans were located in the lower area of the loading plot, separate from most of the other glycans. This assoc-iated was related to the location of several gastric cancer patients distributed in the lower area, which was different from several pancreatic cancer patients on the PC1-PC3 score plot(Supplementary Figure S11C).

 PCA was subsequently performed for each cancer patient group and normal controls using selected glycans increased in each patient group ( Supplementary Figure S12 ). Tentative criteria (*p*-value < 0.05 and fold change ≥3.0; shown in Supplementary Table SIV) were used for inclusion of the increased glycans in each cancer patient group. PCA score plots using these selected glycans showed a tendency to improve segregations of distribution between cancer patients and normal controls (Supplementary Figure S12B, E and H) compared to PCA using total quantified glycans (Supplementary Figure S12A, D and G).

## Discussion

The present study is the first to investigate a wide range of free-glycans in the urine and to assess possible cancer-related elevations in their levels. To understand the features of free-glycans in the urine, detailed structural analyses were performed by HPLC and MS-based methods. Glycans that appeared to be elevated in cancer patients by HPLC were selected, together with some representative glycans unrelated to cancer. These glycans were then analyzed by SRM for inter-specimen comparison.

Urine was found to contain abundant amounts of sialyllactose and sialyl LacNAc trisaccharides, consistent with previous reports (Parkkinen and Finne 1983; Fu and Zopf 1999). In this study, we further identified a number of lactose- and LacNAc-core glycans with modifications of LacNAc-extension, fucose, sulfate and/or sialic acid. Some of these glycans showed cancer-associated elevations in the urine. Enhanced β1,4-galactosyltransferase may be responsible for the increase of lactose and LacNAc. For example, β4GalT-I and β4GalT-V synthesize these disaccharides, albeit with low lactose synthetic activity (van Die et al. 1999), and their increased gene expression in cancer has been reported (Sato and Furukawa 2004; Zhu et al. 2005; Xie et al. 2016; Chatterjee et al. 2019). Lactose-core synthesis may also be promoted by enhanced uptake of glucose in cancer cells (Higashi et al. 1998). Other than sialyllactose trisaccharides, increased glycans frequently possessed α1,3/4-fucose and/or 6-sulfate on the Glc and/or GlcNAc. Some glycans with a combination of fucosylation and sulfation showed elevated levels, although only in a few pancreatic cancer and cholangiocarcinoma patients. These structural features are similar to the previously reported marker candidates found in serum *O*-glycans (Tanaka-Okamoto et al. 2017). This observation suggests that α1,3/4-fucosyltransferases and 6-sulfotransferases might contribute to the elevation of these glycans in addition to the core structure synthesis. Our analyses revealed a marked elevation in the level of a LacNAc-core glycan #100, composed of Lewis X with a sialyl Lewis A extension, in some patients with pancreatic cancer and cholangiocarcinoma. This observed cancer-specificity may be caused by not only the sialyl Lewis A, but also combined type-I and type-II LacNAc backbone (Galβ1-3GlcNAcβ1-3Galβ1-4GlcNAc). Indeed, an enhanced level of this glycan was reported to be associated with cancer in a previous serum *O*-glycan study (Tanaka-Okamoto et al. 2017).

Elevation in the levels of sialylated free-*N*-glycans in cancer patients was observed (#91, #102). The increased amounts of GlcNAc_1_-type glycans match their accumulation in cancer cells and tissues, which supports our assertion that cancer-derived glycans can be detected in urine (Ishizuka et al. 2008; Yabu et al. 2013b). For glycans with the same mono-antennary structure, Man_3_GlcNAc_1_-core show a less dramatic increase in cancer patients than Man_2_GlcNAc_1_-core (#108). This observation suggests that the accumulation of these glycans depends on the degree of degradation. In addition, the mono-antennary glycan with α2,6-sialylation showed an increase than with α2,3-sialylation, which may reflect elevated α2,6-sialyltransferase ST6Gal1 in *N*-glycan synthesis, which is associated with cancer (Pousset et al. 1997; Dall'Olio et al. 2000; Gretschel et al. 2003; Wei et al. 2016). GlcNAc_2_-type free-glycans were also detected but the increased level was less than that observed for GlcNAc_1_-type glycans. Most of the urinary GlcNAc_2_-type glycans probably derived from serum sialylated free-*N*-glycans, which are constantly detectable and resemble hepatic *N*-glycans (Iwatsuka et al. 2013; Seino et al. 2016). Elevation of lactose-core glycans appeared to result from their upregulated synthesis, as described earlier. On the other hand, free-*N*-glycans were degraded products after participated in the protein glycosylation pathway, since the free-*N*-glycans found in this study were complex-type (Suzuki 2016). Sialin, a lysosomal sialic acid transporter may contribute to elevations of the free-*N*-glycans. This transporter induces cytoplasmic accumulation of partially degraded sialylated free-*N*-glycans by translocating them from lysosomes into the cytoplasm in *Autophagy related 5* (*Atg5*) deficient cells (Seino et al. 2013).

 Asialo free-*N*-glycans with β1,6-antennal 6-sulfo-Lewis X showed clear elevated levels in several patients (#83, #105). It was deduced that this specificity was caused by a β1,6-branch, which is initiated by GlcNAcT-V in *N*-glycan synthesis, as well as 3-fucosylation and 6-sulfation of antennal GlcNAc that are similar to the lactose- and LacNAc-core glycans (Dennis et al. 1987; Fernandes et al. 1991; Seelentag et al. 1998). Interestingly, unique free-*N*-glycans with KDN (#106), which had previously been found to accumulate in prostate cancer tissues, did not display elevated levels in the cancer patients analyzed in the present study (Yabu et al. 2013a). The free-*N*-glycan with 4-sulfo-fucose-containing Lewis X (#76) was also a unique structure but showed no cancer related elevation. These unusual structures might be produced as byproducts of other glycans and then excreted due to their resistance to digestion.

Free-glycans corresponding to mucin-type *O*-glycans were also detected. The levels of these mucin-type *O*-glycans did not show a marked elevation in cancer patients, although some core 2-type glycans displayed a slight increase. Therefore, the process by which these glycans are generated is presumably different from lactose- and LacNAc-core glycans as well as free-*N*-glycans. Previous reports detected sialyl core1 as a free-glycan and a serine-*O*-linked glycan in normal human urine, suggesting formation of *O*-glycoprotein-derived free-glycans (Parkkinen and Finne 1983).

Our current research identified acidic Xylα1-3Glc-core glycans. The neutral structures, Xylα1-3Glc and extended Xylα1-3Xylα1-3Glc, were found as urinary free-glycans from healthy individuals (Lundblad and Svensson 1973a; Toyota et al. 1994). These glycans are also known as *O*-Glc glycans on the epidermal growth factor-like domain of glycoproteins such as blood coagulation factors and Notch proteins (Hase et al. 1988; Hase et al. 1990; Moloney et al. 2000). However, NeuAcα2-3Galβ1-4Xylα1-3Glc sequence (#50), which was identified as a glycan with elevated levels in cancer patients, is a novel structure. In addition, a novel structure containing fucosylated Xyl, NeuAcα2-3Galβ1-4(Fucα1-3) Xylα1-3Glc (#60), was also identified in this study. Indeed, #60 showed a particularly high cancer-specific increase in patients with gastric cancer, pancreatic cancer and cholangiocarcinoma. The sequence of NeuAcα2-3Galβ1-4(Fucα1-3) Xyl is structurally similar to sialyl Lewis X and 3′-sialyl-3-fucosyllactose and was presumably formed by upregulation of Lewis-related α1,3-fucosyltransferases in cancer.

 Some of the glycans categorized as other sialylated glycans in this study showed increased levels in cancer patients. The elevation patterns of these glycans were similar to sialylated lactose and LacNAc-core glycans and free-*N*-glycans. The glycans composed of Manα1-6Glc-core with LacNAc-extensions were unique (#88). The LacNAc moiety attached to Man by a β1,2-linkage was similar to *N*-glycans and *O*-Man (Core M1-type) glycans, although the core-disaccharide producing pathway is unknown.

The levels of hexuronylated glycans analyzed in this study showed no major increase in cancer patients. Most hexuronylated glycans appear to be constantly produced by glucuronylation of monosaccharides and small glycans and degradation of glycosaminoglycans. (Stern 2008; Kakizaki et al. 2010; Rowland et al. 2013).

Comparison with existing tumor markers revealed that elevation of free-glycans with sialyl Lewis A was associated with values of CA19-9, of which epitope is also sialyl Lewis A (#100, Figure 6B and Table I). It is noteworthy that free-glycans can serve as an adjunct of existing tumor markers. For example, some gastric cancer patients (G5, 8 and 11) showed lower levels of existing serum tumor markers, CA19-9 and CEA, than cutoff values. However, the levels of #60 in the urine from three patients were clearly elevated and the levels of #50 (Figure 6E), #24, #35, #84, and #118 (Figure 6A) were moderately elevated compared with normal controls. Such cases suggest that urinary free-glycans might be useful markers.

 Because the number of samples was too small, statistical analysis performed in this study was limited to Mann–Whitney test and PCA. To validate the clinical availability of the identified glycans in this paper, a verification study with a larger sample size will be carried out, as previously reported for serum *O*-glycans (Tanaka-Okamoto et al. 2016, 2018a).

In line with previous reports in the literature, the present study compared urinary glycan content based on urinary creatinine concentration (Xia et al. 2013). However, it is known that creatinine levels can vary significantly and cause either overestimation or underestimation in marker measurements, especially in cases of renal impairment (Waikar et al. 2010). A slight increase in the levels of renal function markers, serum creatinine and BUN, were found in some cases including normal controls (in particular N12 and G8). However, no cases with severe renal failure were included in this study (Supplementary Table SI). The glycan levels in N12 were not markedly different from those of the other normal controls. Therefore, we believe creatinine correction can be considered a reliable criterion in this study. However, the relationship between urinary creatinine level and the amounts of free-glycans remains unclear. Further research into a more reliable standard is required.

Elevated levels of free-glycans may also be associated with other diseases, inflammation or pregnancy. For example, a previous study indicated that patients with rheumatoid arthritis exhibited elevated levels of urinary sialyllactose and sialyl LacNAc (Maury 1978). Thus, to better understand the relationship between free-glycans and cancer it is also important to study glycan production in tumor tissues and cultured cells.

Future studies to identify highly specific glycan markers will need to focus on cancer-related structural features, such as branching, backbone extension, fucosylation, sulfation and acetylation. The amount of urine used in this study corresponded to 16 μg of urinary creatinine for RP-HPLC and 8 μg of urinary creatinine for SRM analysis (if calculated as 100 mg/dL creatinine urine, 16 μL and 8 μL, respectively). The advantage of urine from an analytical perspective is that samples can be easily obtained in large quantities by noninvasive means. Thus, large-scale preparation and analysis of urine free-glycans focused on characteristic glycans that are present at low concentrations may help identify specific marker candidates.

## Materials and methods

### Urine samples

Urine samples of patients with gastric cancer (*n* = 13, male 8, female 5, mean age 67.0 years), pancreatic cancer (*n* = 10, male 6, female 4, mean age 62.7 years) and cholangiocarcinoma (*n* = 4, male 2, female 2, mean age 68.0 years) were obtained from Osaka International Cancer Institute. The clinical features of the 27 patients examined in this study are summarized in Table I. Additional information for some patients is also shown in Supplementary Table SI. The patients were numbered G1–G13 for gastric cancer patients, P1–P10 for pancreatic cancer patients and C1–C4 for cholangiocarcinoma patients. Urine samples of normal controls (*n* = 21, male 15, female 6, mean age 63.2 years) were obtained from cancer-free healthy volunteers. This study was approved by the Local Ethics Committee of Osaka International Cancer Institute. Informed consent was obtained from each patient and volunteer.

### Isolation of free-glycans from urine samples

Urinary creatinine concentrations were determined using a Determiner-L CRE kit (Kyowa Medex, Tokyo, Japan). Each value is given in Table I. To normalize the concentration of urine, volumes used for sample preparation were corrected by creatinine concentration. Urine samples equivalent to 400 μg creatinine were used as starting material. Urine samples were loaded onto a column packed with 1 mL of Dowex 50 W-X8 (H^+^-form, 200–400 mesh, FUJIFILM Wako Pure Chemical, Osaka, Japan) and washed using 4 mL of water. Flow-through and wash fractions were collected as free oligosaccharide containing fractions. The eluates were neutralized by addition of 0.2 mL of saturated sodium bicarbonate. Eluates were further applied to a graphite carbon cartridge (InertSepGC 300 mg; GL Science, Tokyo, Japan). The column was washed with 3 mL water and oligosaccharides were subsequently eluted with 3 mL of 60% acetonitrile in 50 mM ammonium acetate, pH 7.0, and lyophilized.

### Preparation and separation of PA-glycans from human urine

Experimental procedures, such as preparation and separation of PA-glycans, were performed as previously reported (Tanaka-Okamoto et al. 2016). Briefly, reducing ends of the free oligosaccharides were labeled with a fluorophore, 2-aminopyridine, by reductive amination (Hase et al. 1978; Kuraya and Hase 1992). Excess reagents were subsequently removed by phenol/chloroform extraction and cation exchange chromatography. PA-glycans were further purified using a graphite carbon cartridge (InertSepGC 150 mg; GL Science) (Natsuka et al. 2011). Finally, the eluted PA-glycans were lyophilized and dissolved in 800 μL water.

PA-oligosaccharides were separated on a Shimadzu LC-20A HPLC system (Shimadzu, Tokyo, Japan) equipped with a Waters 2475 fluorescence detector. The following experimental procedures, including normal phase HPLC and reversed phase HPLC have been reported previously (Misonou et al. 2009). Normal phase HPLC was performed on a TSKgel Amide-80 column (5 μm, 2 × 250 mm; Tosoh, Tokyo, Japan). Reversed phase HPLC was performed on a TSKgel ODS-80Ts or ODS-80Ts-QA column (5 μm, 2 × 150 mm; Tosoh). The elution position of each PA-glycan was normalized into glucose units (GU) based on the elution positions of PA-isomaltooligosaccharides. Thus, a given compound on these two columns provides a unique set of GU (NP) and GU (RP) values, which correspond to coordinates on the two-dimensional map. Weak anion exchange HPLC was performed at 30°C on a TSKgel DEAE-5PW column (10 μm, 7.5 × 75 mm; Tosoh). The glycans eluted from NP- and RP- HPLC were collected in 1.5-mL tubes or 96-well plates using a fraction collector, 222XL liquid handler (Gilson, Middleton, WI). The glycans eluted from anion exchange HPLC were collected in 15-mL tubes using a FC 204 fraction collector (Gilson). HPLC conditions used for the separation of PA-Hex are presented in Supplementary Figure S1.

### Glycosidase digestion

Unless otherwise noted, glycosidases supplied as solution were used at 1/6–1/10 dilution in 1× GlycoBuffer 1 (50 mM sodium acetate and 5 mM CaCl_2_ pH 5.5; New England Biolabs, Ipswich, MA) and digestions were performed at 37°C overnight (more than 16 h). *X. manihotis* β-galactosidase and α-1,2-fucosidase, *S. pneumoniae* β-*N*-acetylglucosaminidase *S. plicatus* β1,3/4/6-*N*-acetylhexosaminidase and *E. meningoseptica* α-*N*-acetylgalactosaminidase were from New England Biolabs. *S. pneumoniae* β-galactosidase was from ProZyme (San Leandro, CA) and bovine kidney α-fucosidase was from Sigma-Aldrich (St Louis, MO). Lacto-*N*-biosidase and α1,3/4-fucosidase isolated from *Streptomyces* sp. 142 were purchased from Takara Bio (Shiga, Japan). Neuraminidase from *S. typhimurium* (Takara Bio) was used at 1.7 mU/μL for 2 h to maintain specificity at the nonreducing terminal α2,3-linkage, and at 8.3 mU/μL overnight for nonreducing terminal α2,3/6-linkages (Shida et al. 2009). The neuraminidase from *Arthrobacter ureafaciens* (Nacalai, Kyoto, Japan) was used at a concentration of 2 mU/μL. Sodium acetate (50 mM pH 4.5) was used for β-glucronidase from *P. vulgata* (Sigma-Aldrich), β-galactosidase from jack bean (Seikagaku Corp, Tokyo, Japan) and α-mannosidase from jack bean (New England Biolabs). Sodium citrate (50 mM pH 4.5) was used for β-*N*-acetylhexosaminidase from human placenta (Sigma-Aldrich). Sodium phosphate (100 mM pH 6.8) was used for β-galactosidase from *E. coli* (FUJIFILM Wako Pure Chemical, Tokyo, Japan) and α-xylosidase from *E. coli* (Megazyme, Bray, Ireland), and the former enzyme (supplied as powder) was used at 1.5 U/μL. Coffee bean α-galactosidase (Sigma-Aldrich) was incubated in sodium phosphate (100 mM pH 6.4) at 30°C. For glycosidase digestions, an aliquot of each HPLC-purified glycan was used. The digested glycans were fractionated by RP-HPLC for separation of products from undigested glycans and other glycans. The glycans were analyzed by mass spectrometry to confirm digestion and then subjected to NP-HPLC for 2D-HPLC mapping.

### Methanolysis of sulfated PA-glycans

PA-glycans were methanolyzed with 10 μL of 50 mM HCl in methanol at 37°C for 3 h (Slomiany et al. 1981; Murakami et al. 2007). After the reaction, the products were evaporated to dryness three times with an additional 100 μL of 2-propanol. The methanolyzed glycans were then analyzed in the same way as the glycosidase-digests.

### Periodate oxidation and reduction of PA-glycans

PA-glycans were treated with 80 mM sodium periodate in 50 mM sodium acetate buffer at 4°C for 2 days in the dark. The reaction was stopped by addition of ethylene glycol and the oxidized glycans were then reduced by 100 mM sodium borohydride at room temperature for 1 h (Irimura et al. 1981; Omichi and Hase 1995; Minamida et al. 1996). The product glycans were desalted by graphite carbon cartridge and analyzed by ESI-MS and MS^n^.

### Mass spectrometry for structural analysis

Mass spectrometry for structural analysis was carried out using a ESI-MS on a LTQ XL linear ion trap mass spectrometer (Thermo Scientific, San Jose, CA) connected to a Paradigm MS4 HPLC system (Michrom BioResources, Auburn, CA). Spray voltages were set to 3 kV and −2 kV for positive and negative mode, respectively. The temperature of the ion source was maintained at 250°C. Temperature of the capillary was set to 300°C. Sheath gas was set to 40 units. Settings for the tube lens voltage and range for a full MS scan varied from sample to sample. MS^n^ was performed by a data-dependent mode or selected parent ion isolation. The PA-glycans were trapped on an InertSustain AQ-C18 column (3 μm, 1 × 50 mm; GL sciences) equilibrated with 5 mM acetic acid titrated to pH 6.0 with triethylamine and then subsequently eluted with 50% (v/v) acetonitrile. HPLC was performed using a flow rate of 50 μL/min at room temperature.

### Mass spectrometry for quantification by SRM

Mass spectrometry for quantification by SRM was performed using a 4500 Q Trap hybrid triple quadrupole/linear ion trap mass spectrometer (Applied Biosystems, Framingham, MA) connected to a Shimadzu LC-20A HPLC system (Shimadzu). For the HPLC, column and solvents were the same as used for the structural analyses. Data acquisition was performed with an ion spray voltage of 4.5 kV, curtain gas of 20 psi, nebulizer gas (GS1) of 30 psi, turbo gas (GS2) of 40 psi, an interface heater temperature of 350°C and dwell time of 50 ms. Two SRM transitions were monitored and acquired at low resolution both in the first and third quadrupoles (Q1 and Q3). The values of Q1 and Q3 are shown in Supplementary Table SIII. SRM data acquired on the 4500 QTRAP were analyzed by MultiQuant v2.1.1 (Applied Biosystems). Integration settings included a smoothing width of one point and a peak splitting factor of 2. The more abundant transition of the two transitions was selected as the quantifier transition to be used in quantitative analyses. The peak area in the extracted ion chromatogram (XIC) of each SRM transition was measured. Based on the observation of signal-to-noise ratio of XIC corresponding to transitions monitored for PA-glycans, we used an intensity of 150 as the LOQ (limit of quantification).

### Statistical analysis

 For the data of the glycan levels obtained from SRM or fluorescence detection, Mann–Whitney *U* test was performed in GraphPad PRISM version 6.0. PCA was performed using the web-based platform, MetaboAnalyst version 4.0 (  Chong et al. 2019 , Xia et al. 2009). The values lower than LOQ were set to 0. Before submission to PCA, the data of the glycan levels were autoscaled.

## Funding

This work was supported in part by Grant-in-Aid for Scientific Research (C) no. 18K07433 from the Ministry of Education, Culture, Sports, Science and Technology of Japan.

This work was supported in part by a medical research grant from the Osaka Medical Research Foundation for Intractable Diseases.

## Conflict of interest statement

None declared.

## Abbreviations

AU, arbitrary units; BUN, blood urea nitrogen; DEAE, diethylaminoethyl; ESI, electrospray ionization; GU, glucose units; HexA, hexuronic acid; HPLC, high performance liquid chromatography; KDN, 2-keto-3-deoxy-nonulosonic acid; MS, mass spectrometry; MS^n^, multistage mass spectrometry; NP, normal phase; PA, pyridylamino- or 2-aminopyridine; PCA, principal component analysis; Pen, pentose; RP, reversed phase; SRM, selected reaction monitoring; Tal, talose; TalNAc, *N*-acetyltalosamine; TEA, triethylamine.

## Supplementary Material

Supplementary_Data_rev2_Hanzawa_et_al_cwaa100
