# Supplementary material for: Investigation of acidic free-glycans in urine and their alteration in cancer
Source: Glycobiology. 2020 Oct 29;31(4):391–409. doi: 10.1093/glycob/cwaa100 (PMC8091460; doi:10.1093/glycob/cwaa100)
Supplement: Supplementary_Data_rev2_Hanzawa_et_al_cwaa100 [file supplementary_data_rev2_hanzawa_et_al_cwaa100.pdf]

Supplementary Data

Investigation of acidic free-glycans in urine and their alteration in cancer

Fig. S1. HPLC analysis of reducing terminal Hex-PA.

Fig. S2. Structural analysis of glycan #68, containing a β-galactosyllactose backbone by two-dimensional HPLC mapping.

Fig. S3. Structural analysis of the lactose- and LacNAc-core glycans containing fucosylated and sulfated galactose by two-dimensional HPLC mapping and MS^2 after periodate treatment.

Fig. S4. Analysis of LacNAc-extension with 6-sulfate group by two-dimensional HPLC mapping and MS^n after periodate treatment.

Fig. S5. MS^n analysis of the fucosylated and sulfated free-*N*-glycan with fucosidase-resistance after periodate treatment.

Fig. S6. Structural analysis of the free-*N*-glycan, NeuAcα2-6Galβ1-4GlcNAcβ1-4GlcNAc-PA by two-dimensional HPLC mapping and MS^2 after periodate treatment.

Fig. S7. Structural analysis of sialylated glycans containing an unusual Manα1-6Glc-core by two-dimensional HPLC mapping and MS^2 after periodate treatment.

Fig. S8. Structural analysis of the glucuronylated glycans with α1,4-GalNAc-capping by two-dimensional HPLC mapping and MS^2 after periodate treatment.

Fig. S9. Supplementary data of levels of representative free-glycans in the urine from SRM analysis.

Fig. S10. Supplementary data of levels of representative free-glycans in the urine from HPLC-fluorescent detection.

Fig. S11. Principal component analysis (PCA) plots using glycan levels of cancer patients and normal controls.

Fig. S12. Principal component analysis (PCA) of the glycans increased in cancer patients.

Table SI. Clinical information of the normal controls and patients with gastric cancer, pancreatic cancer and cholangiocarcinoma (supplementary).

Table SII. Estimated structures of acidic free-glycans from urine (supplemental).

Table SIII. Q1 and Q3 values for SRM measurements of the PA-glycans.

Table SIV. Comparison between cancer patient groups and normal controls for each glycan level.

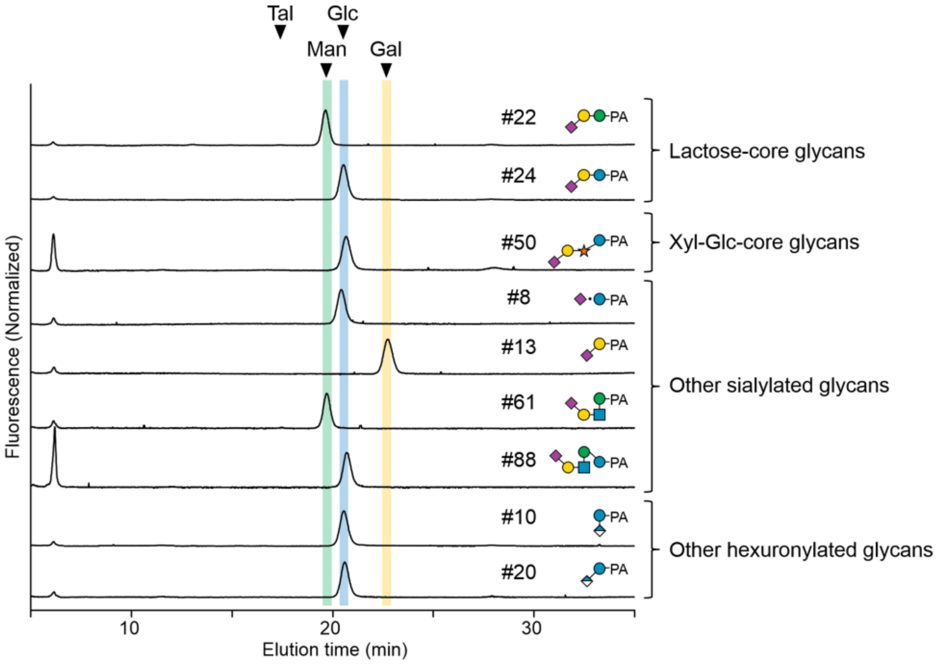

Fig. S1. HPLC analysis of reducing terminal Hex-PA. After digestion into the reducing terminal monosaccharide, each PA-glycan was analyzed by HPLC for separation of PA-hexoses. Arrowheads indicate standard PA-labeled hexoses.

HPLC separation was performed using on a Shimadzu LC-20A HPLC system equipped with a Waters 2475 fluorescence detector. The column was a TSKgel Amide-80 (3µm, 3 × 150 mm; Tosoh) operated at a flow rate of 0.2 mL/min and column temperature of 40°C. The solvents used were (A) acetonitrile and (B) 0.2% (*v/v*) formic acid adjusted to pH 4.4 with aqueous ammonia. The column was equilibrated at 12% of solvent (B) and held for 28 min after sample injection (PA-monosaccharides were eluted isocratically). The column was washed by linearly increasing the amount of solvent (B) to 40% in 5 min, which was then held for 2 min prior to re-equilibration for 30 min. The wavelength settings for the fluorescence detector were as follows: excitation 315 nm, emission 400 nm.

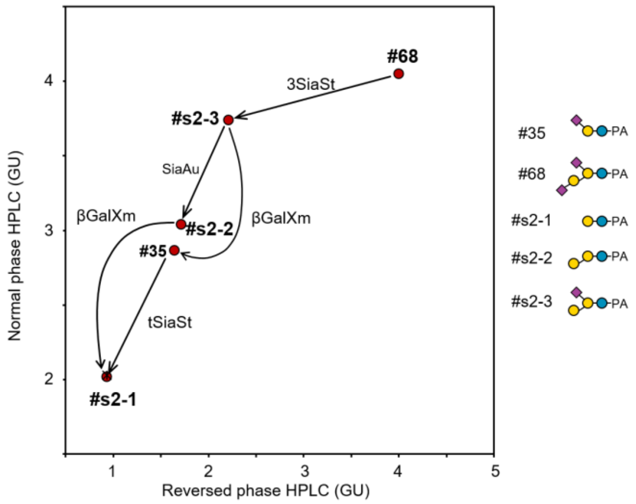

Fig. S2. Structural analysis of glycan #68, containing a β-galactosyllactose backbone by two-dimensional HPLC mapping. The standardized elution positions are shown. Red circles and asterisks indicate the positions of the sample glycans and standard glycans, respectively. Solid arrows indicate shifts of the glycans by glycosidases. The glycosidases used are indicated as follows: 3SiaSt, α-neuraminidase under the condition for non-reducing terminal α2,3-linkages (from *S. typhimurium*); tSiaSt, α-neuraminidase for non-reducing terminal α2,3/6-linkages (from *S. typhimurium*); SiaAu, α-neuraminidase with broad specificity (from *A. ureafaciens*); βGalXm, β-galactosidase with specificity for β1,3>6>4 (from *X. manihotis*). The PA-glycan #68, composed of Hex_3NeuAc_2-PA, *m/z* 1165 $[M+H]^+$, was completely de-sialylated by SiaAu resulting in Hex_3-PA. This trisaccharide showed resistance to digestion with *S. pneumoniae* β1,4-galactosidase (not shown), but was digested by βGalXm into lactose. However, one Sia residue was removed by 3SiaSt, and sequential βGalXm treatment produced 6'-sialyllactose. These results suggest that #68 contained β3'-galactosyl lactose as a neutral backbone, which is found in milk oligosaccharides, with 3'',6'-disialylation.

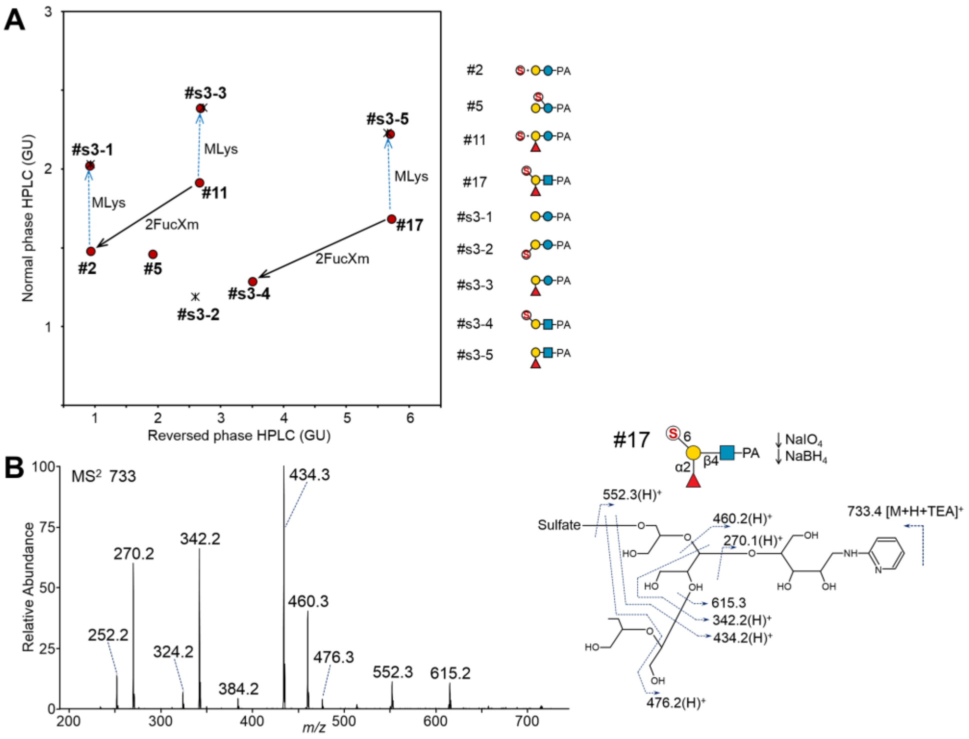

Fig. S3. Structural analysis of the lactose- and LacNAc-core glycans containing fucosylated and sulfated galactose by two-dimensional HPLC mapping and MS^2 after periodate treatment. (A) Two-dimensional HPLC mapping analysis. The standardized elution positions are shown. Red circles and asterisks indicate the positions of the sample glycans and standard glycans, respectively. Solid arrows labeled with "2FucXm" indicate shifts by α1,2-fucosidase (from *X. manihotis*). Dotted arrows with "M-Lys" indicate shifts resulting from methanolysis. Glycans #11 and #17 were susceptible to 2FucXm. Glycan #2 corresponded to the de-fucosylated form of #11 and was resistant to β-galactosidase from *E. coli* (data not shown). By methanolysis, #11 and #17 were shifted to 2'-fucosyllactose and 2'-fucosyl LacNAc, respectively. (B) For glycan #17, MS^2 analysis was performed after treatment with sodium periodate and then sodium borohydride to obtain linkage information. MS^2 spectrum of the product from the protonated ion with triethylamine at *m/z* 733 $[M+H+TEA]^+$ ($Hex_1HexNAc_1dHex_1Sulfate_1$-PA $+2\times2H$ $-2\times CH_2O$) in positive mode MS suggested 2'-fucosylation and 6'-sulfation, respectively.

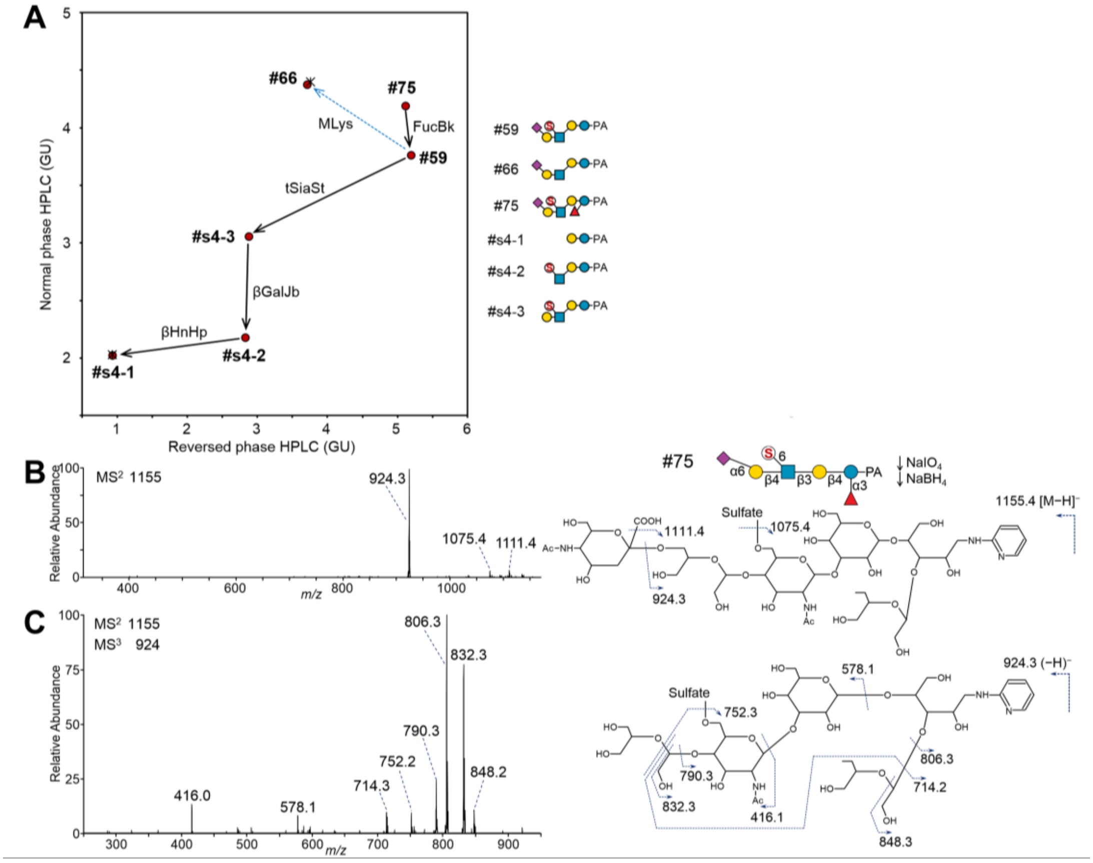

Fig. S4. Analysis of LacNAc-extension with 6-sulfate group by two-dimensional HPLC mapping and MS^n after periodate treatment. (A) Two-dimensional HPLC mapping analysis. The standardized elution positions are shown. Red circles and asterisks indicate the positions of the sample glycans and standard glycans, respectively. Solid arrows indicate shifts of the glycans by glycosidases. Dotted arrows indicate shifts resulting from methanolysis. The glycosidases used are indicated as follows: tSiaSt, α-neuraminidase (*S. typhimurium*) for non-reducing terminal α2,3/6-linkages; FucBk, α-fucosidase (bovine kidney); βGalJb, β-galactosidase (jack bean); bHnHp, β-*N*-acetylhexosaminidase (human placenta). After de-sialylation, the antennal Galβ1-4(Sulfate-6)GlcNAcβ-structure was sequentially digested with βGalJb and bHnHp. This structure showed resistance to digestion with β-galactosidases and β-*N*-acetylhexosaminidases from other organisms used in this study (data not shown). (B and C) To obtain the linkage information, MS^n analysis was performed after treatment with sodium periodate and subsequently sodium borohydride. Glycan #75 is shown as a representative example. (B) MS^2 spectrum of the product from the de-protonated ion at *m/z* 1155 $[M-H]^-$ ($Hex_3HexNAc_1dHex_1NeuAc_1Sulfate_1$-PA +2×2H, −5×$CH_2O$) in negative mode MS. (C) MS^3 spectrum of the de-sialylated Y-fragment ion at *m/z* 924 from MS^2.

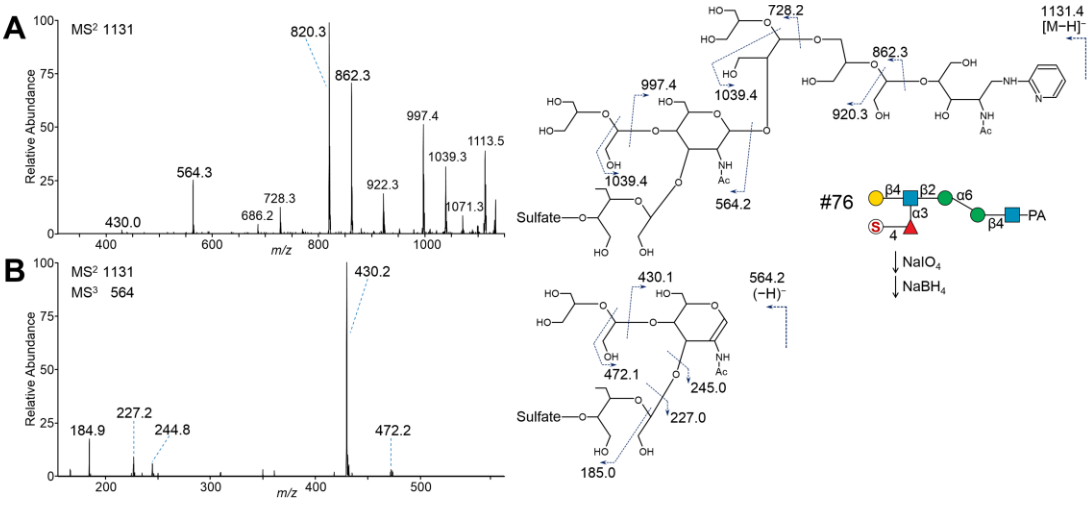

Fig. S5. MS^n analysis of the fucosidase-resistant fucosylated and sulfated free-*N*-glycan after periodate treatment. To obtain the linkage information, the glycan #76 (Figure 3, Fr 6), composed of $Hex_3HexNAc_2dHex_1Sulfate_1$-PA at *m/z* 1316 $[M+H+TEA]^+$, was treated with sodium periodate and subsequently with sodium borohydride and then analyzed by MS^n. (A) MS^2 spectrum of the product from the de-protonated ion at *m/z* 1131 $[M-H]^-$ ($Hex_3HexNAc_2$ $dHex_1Sulfate_1$-PA +4×2H, −3×CH_2O) in negative mode MS. An ion at m/z 564 was consistent with an antennal structure produced by loss of cleaved product of Manα1-6Manβ1-4GlcNAc-PA. (B) MS^3 of the antennal B-ion at *m/z* 564. The base peak ion at m/z 430 indicated loss of Hex residue (two C-C bonds cleaved, 134 Da). Ions at *m/z* 227 and 245 were B- and C-type ions of a sulfated dHex residue with one C-C bond cleavage and smaller ion at *m/z* 185, which corresponded to a C_4 fragment of C-3 to C-6 carbons with a sulfate group at C-4 position, indicating a 4-sulfo-Fuc residue.

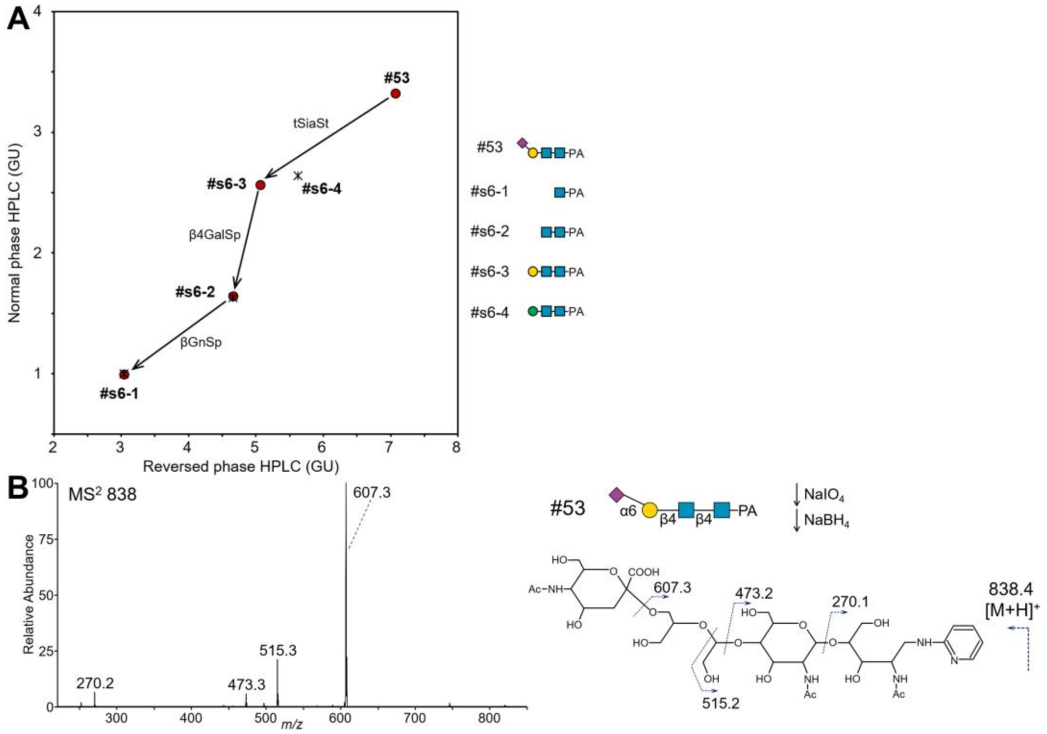

Fig. S6. Structural analysis of the free-*N*-glycan, NeuAcα2-6Galβ1-4GlcNAcβ1-4GlcNAc-PA by two-dimensional HPLC mapping and MS^2 after periodate treatment. (A) Two-dimensional HPLC mapping analysis. The unusual free-*N*-glycan with GlcNAc$_2$-core (#53) was analyzed. The standardized elution positions are shown. Red circles and asterisks indicate the positions of the sample glycans and standard glycans, respectively. Solid arrows indicate shifts of the glycans by glycosidases. The glycosidases used in this analysis are indicated as follows: tSiaSt, α-neuraminidase for non-reducing terminal α2,3/6-linkages (*S. typhimurium*); βGalSp, β1,4-galactosidase (*S. pneumoniae*); βGnSp, β-*N*-acetyl-glucosaminidase (*S. pneumoniae*). Glycan #53 was digested with *S. typhimurium* neuraminidase under the conditions for α2,3/6-linkages but not α2,3-linkages. The desialylated trisaccharide (#s6-3) did not correspond to the general *N*-glycan core, Manβ1-4GlcNAcβ1-4GlcNAc-PA (#s6-4), and was digested by *S. pneumoniae* β1,4-galactosidase into *N*,*N*'-diacetylchitobiose (#s6-2). (B) MS^2 spectrum of the periodate-cleaved product from the protonated ion at *m/z* 838 [M+H]$^+$ (Hex$_1$HexNAc$_2$NeuAc$_1$-PA +2H −4×CH$_2$O) in positive mode MS. This spectrum was consistent with the NeuAc2-3Hex1-3/4HexNAc1-4HexNAc-PA sequence, supporting the result obtained from the glycosidase digestions.

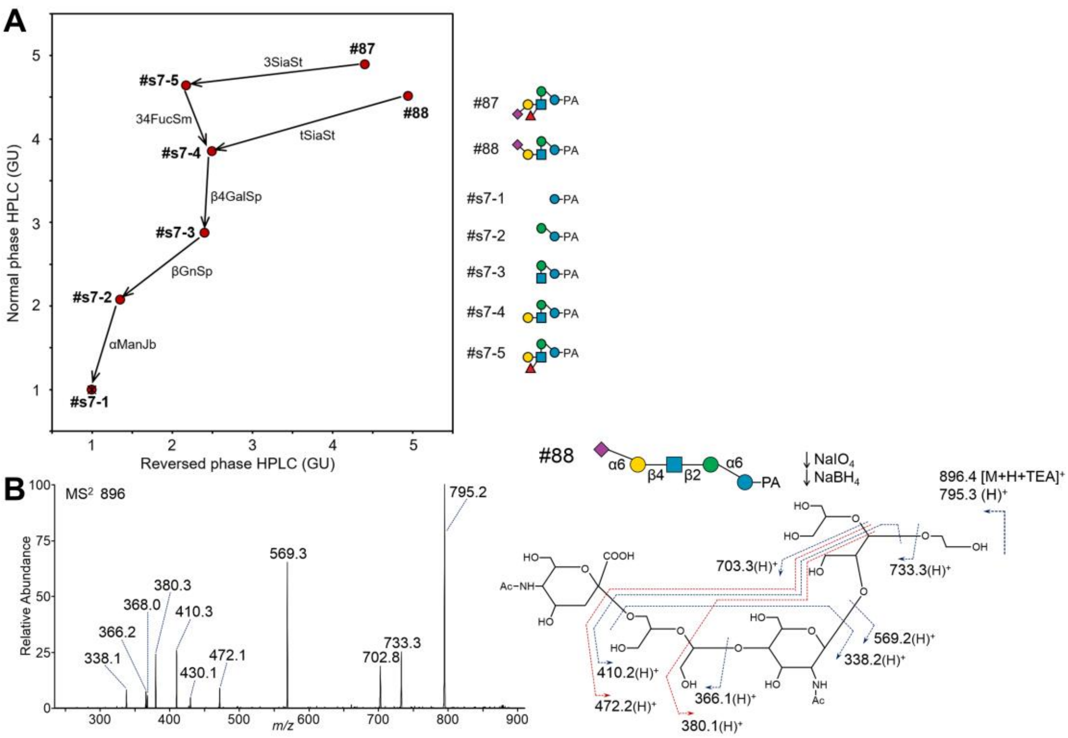

Fig. S7. Structural analysis of sialylated glycans containing an unusual Galβ1-4GlcNAcβ1-2Manα1-6Glc backbone by two-dimensional HPLC mapping and MS^2 after periodate treatment. The glycans #87 and #88, containing a $Hex_3HexNAc_1$-PA backbone, which did not match any lactose-core glycan were analyzed. (A) Two-dimensional HPLC mapping analysis. The standardized elution positions are shown. Red circles and asterisks indicate the positions of the sample glycans and standard glycans, respectively. Solid arrows indicate shifts of the glycans by glycosidases. The glycosidases used are indicated as follows: 3SiaSt, α-neuraminidase under the condition for non-reducing terminal α2,3-linkages (*S. typhimurium*); tSiaSt, α-neuraminidase for non-reducing terminal α2,3/6-linkages (*S. typhimurium*); βGalSp, β1,4-galactosidase (*S. pneumoniae*); 34FucSm, α1,3/4-fucosidase (*Streptomyces* sp. 142); βGnSp, β-*N*-acetylglucosaminidase (*S. pneumoniae*); αManJb, α-mannosidase (jack bean). The HexNAc residue showed resistance to *S. plicatus* β1,3/4/6-*N*-acetylhexosaminidase (data not shown). The reducing terminal Hex was also confirmed by monosaccharide HPLC, shown in Figure S1. (B) MS^2 spectrum of the periodate-cleaved product from the protonated ion with triethylamine at *m/z* 896 $[M+H+TEA]^+$ ($Hex_2HexNAc_1NeuAc_1$-$C_2H_6O_2$ +2×2H −3×CH_2O) in positive mode MS. The PA-moiety of the glycan was lost, due to cleavage between C-4 and C-5 of the reducing terminal Glc-PA residue.

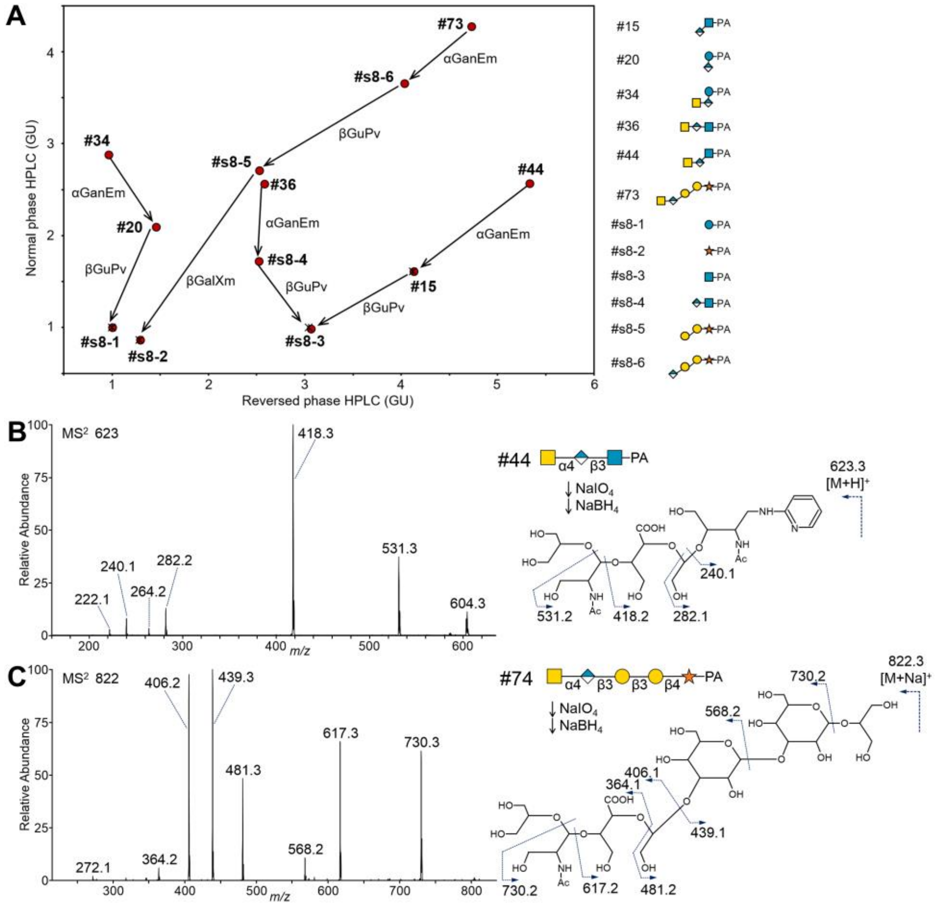

Fig. S8. Structural analysis of the glucuronylated glycans with α1,4-GalNAc-capping by two-dimensional HPLC mapping and MS[2] after periodate treatment. (A) Two-dimensional HPLC mapping analysis. Glycans containing a HexNAc-HexA-structure at their reducing termini (#34, #36, #44, #74) were sequentially digested by glycosidases. These glycans showed susceptibility to digestion with α-*N*-acetylgalactosaminidase. The standardized elution positions are shown. Red circles and asterisks indicate the positions of the sample glycans and standard glycans, respectively. Solid arrows indicate shifts of the glycans by glycosidases. The glycosidases used are indicated as follows: αGanEm, α-*N*-acetylgalactosaminidase (*E. meningoseptica*); βGuPv, β-glucuronidase (*P. vulgata*); βGalXm, β-galactosidase with specificity for β1,3>6>4 (*X. manihotis*). (B and C) To obtain the linkage information, MS[2] analysis was performed after treatments with sodium periodate and sodium borohydride. Glycans #36 and #74 are shown as representative examples. (B) MS[2] spectrum of the product of #36 from protonated ion at *m/z* 623 $[M+H]^+$ ($HexNAc_2HexA_1$-PA +2×2H −2×CH_2O) in positive mode MS. (C) MS[2] spectrum of the product of #74 from the sodiated ion at *m/z* 822 $[M+Na]^+$ ($Hex_2HexNAc_1HexA_1$ +$C_3H_8O_3$ +2×2H) in positive mode MS. The PA-moiety of the glycan was lost, due to the cleavage between C2 and C3 of the reducing terminal Xyl-PA residue.

(Figure S9)

A Lactose-core glycans (Supplemental)

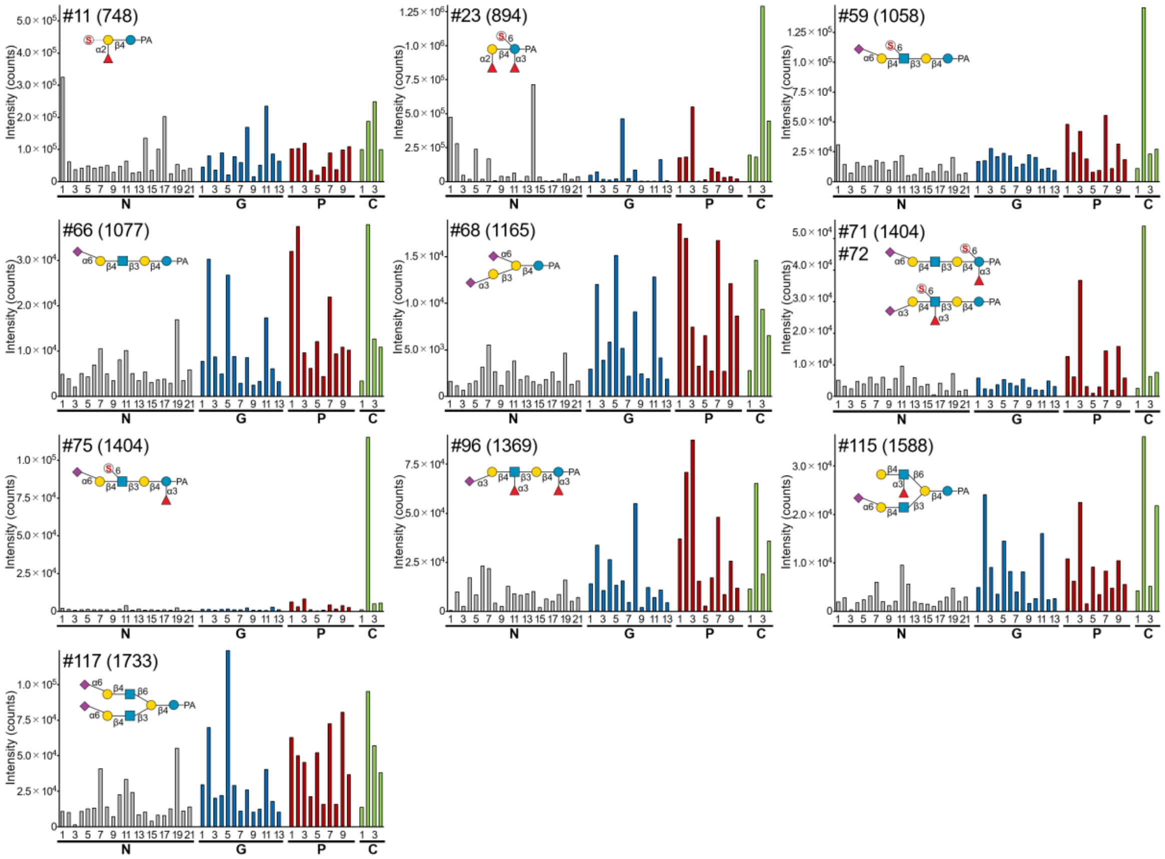

B LacNAc-core glycans (Supplemental)

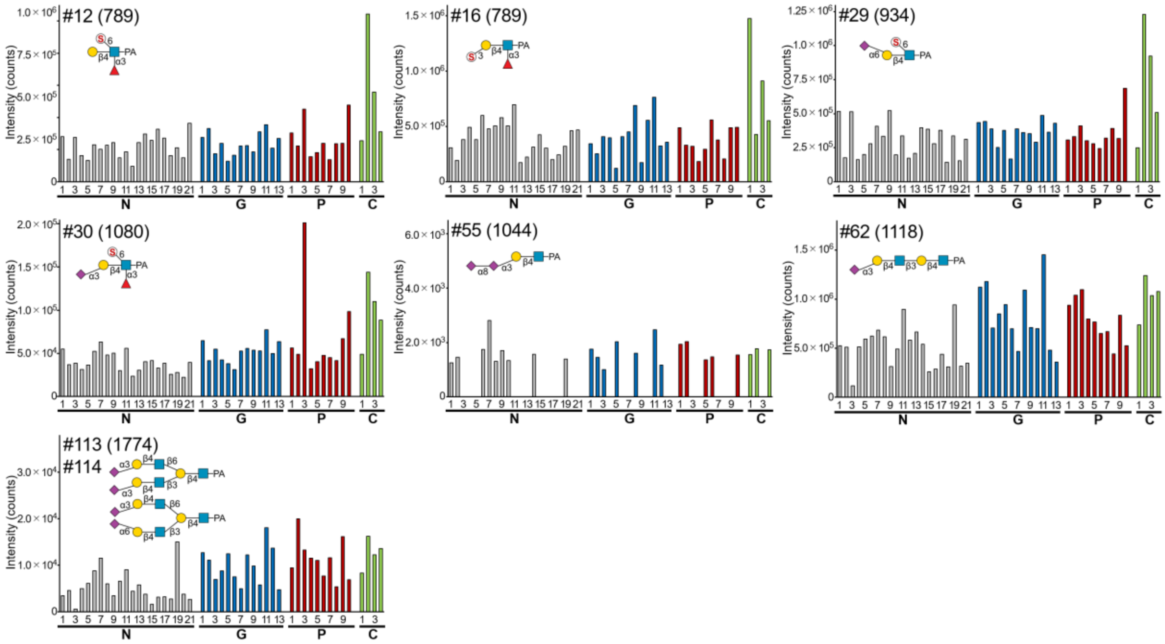

(Figure S9, continued)

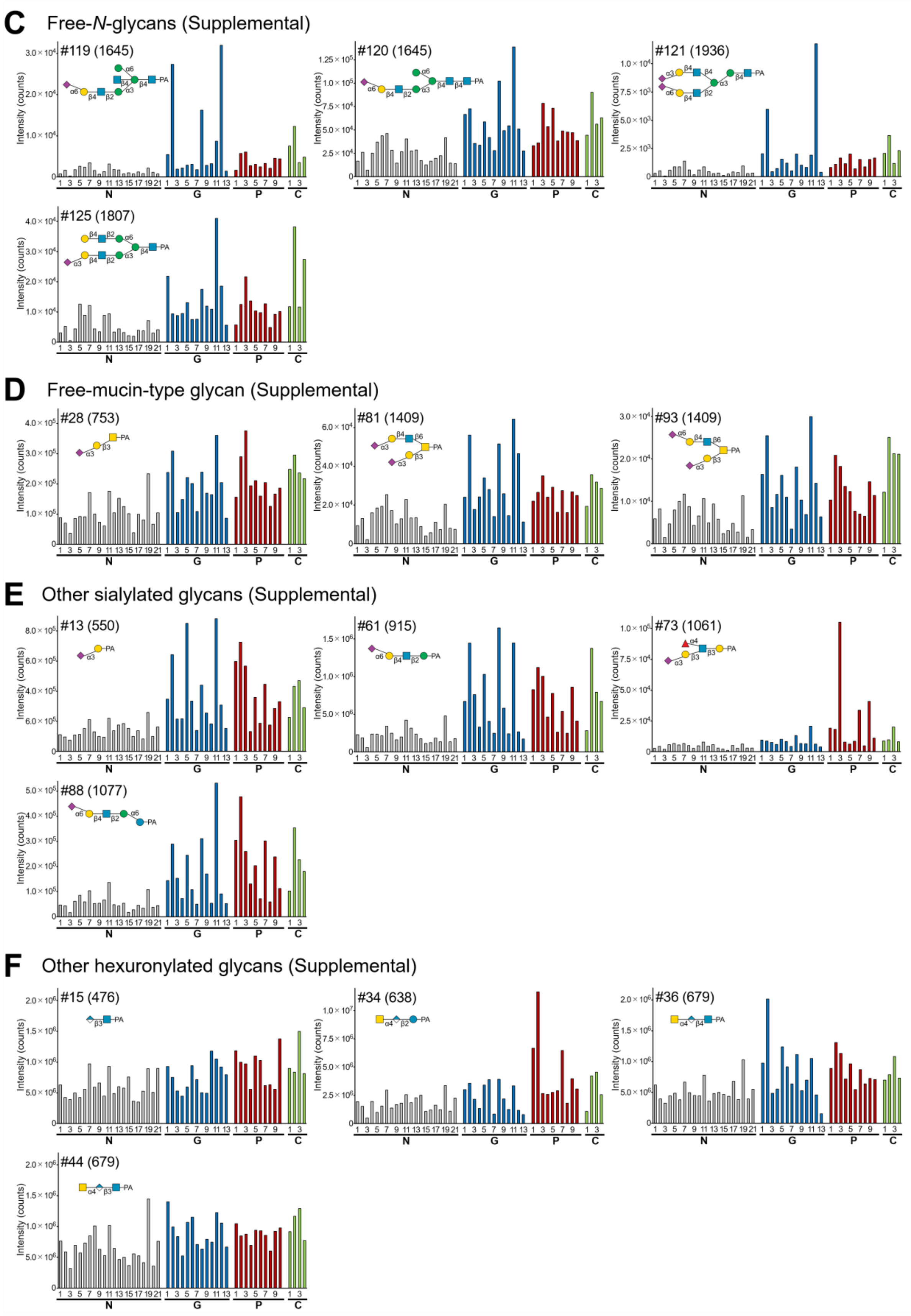

Fig. S9. Supplementary data of levels of representative free-glycans in the urine from SRM analysis. The amount of urine sample corresponded to 8 μg of creatinine, but exceptionally 0.8 μg of creatinine for glycans #13, #15 and #28. The peak areas in the extracted ion chromatogram (XIC) of SRM measurements are shown. The levels of the glycans are indicated by bars as follows: normal controls (N1–N21), gray; gastric cancer patients (G1–G13), blue; pancreatic cancer patients (P1–P10), red; and cholangiocarcinoma patients (C1–C4), light green. In each glycan panel, glycan number and estimated structure are indicated. (A) Lactose-core glycans #11, #23, #59, #66, #68, mixture of #71 and #72, #75, #96, #115 and #117 are shown. (B) LacNAc-core glycans #12, #16, #29, #30, #55, #62 and mixture of #113 and #114 are shown. (C) Free-*N*-glycans #119–121 and #125 are shown. (D) Mucin-type free-glycans. The glycans #28, #81 and #93 are shown. (E) Other sialylated glycans #13, #61, #73 and #88 are shown. (F) Other hexuronylated glycans #15, #34, #36 and #44 are shown.

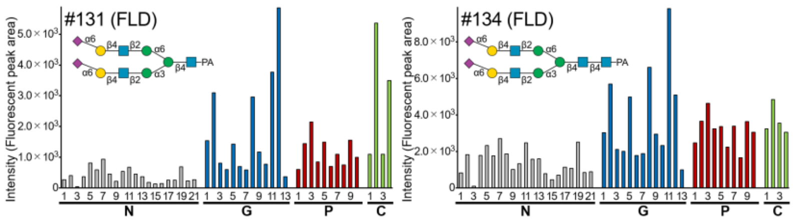

Fig. S10. Supplementary data for the levels of representative free-glycans of in the urine from HPLC analyses obtained by a fluorescent detector (FLD). The peak areas obtained from the RP-HPLC chromatograms (Figure 3) are shown. Levels of the glycans are indicated by bars as follows: normal controls (N1–N21), gray; gastric cancer patients (G1–G13), blue; pancreatic cancer patients (P1–P10), red; and cholangiocarcinoma patients (C1–C4), light green. In each glycan panel, glycan number and estimated structure are indicated.

(Figure S11)

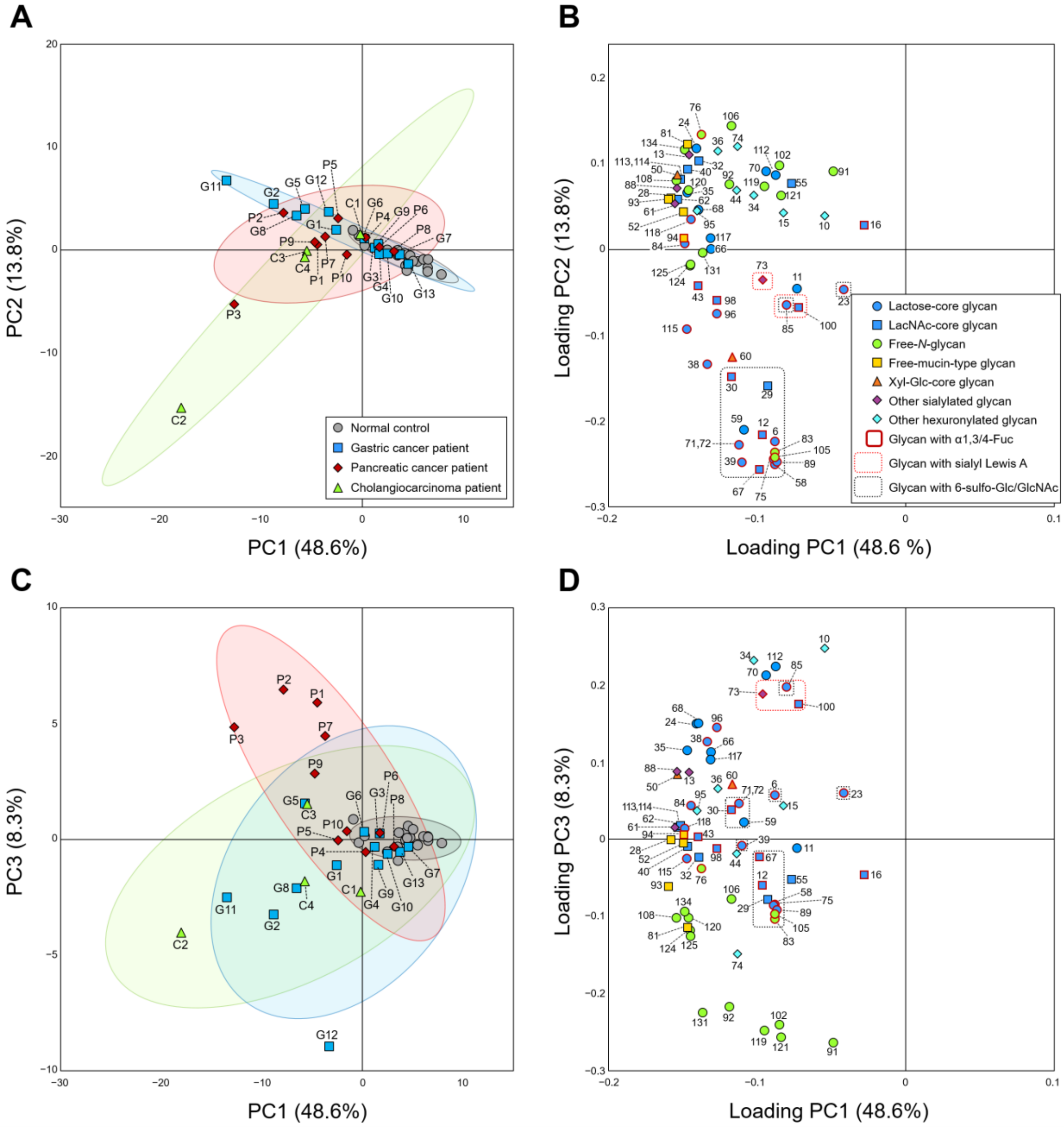

A
B
C
D
PC1 (48.6%)
PC2 (13.8%)
PC3 (8.3%)
Loading PC1 (48.6 %)
Loading PC1 (48.6%)
Loading PC2 (13.8%)
Loading PC3 (8.3%)
Normal control
Gastric cancer patient
Pancreatic cancer patient
Cholangiocarcinoma patient
Lactose-core glycan
LacNAc-core glycan
Free-N-glycan
Free-mucin-type glycan
Xyl-Glc-core glycan
Other sialylated glycan
Other hexuronylated glycan
Glycan with α1,3/4-Fuc
Glycan with sialyl Lewis A
Glycan with 6-sulfo-Glc/GlcNAc

Fig. S11. Principal component analysis (PCA) plots using glycan levels of cancer patients and normal controls. PCA was performed using the data of glycan levels obtained from SRM or fluorescent detection (Figure 6, S9 and S10) in MetaboAnalyst 4.0 software. Plots were generated by Microsoft Excel 2013 for detailed labeling of the symbols. (A) Score plot of PC1 to PC2. (B) Loading plot of PC1 to PC2. (C) Score plot of PC1 to PC3. (D) Loading plot of PC1 to PC3. (A and C) Symbols indicate the cases as follows: gray circle, normal control; blue square, gastric cancer patient; red diamond, pancreatic cancer patient; green triangle, cholangiocarcinoma patient. The ellipses correspond to 95% confidence region of each group. (B and D) The glycans are plotted as symbols based on their structures, as follows; blue circle, lactose-core; blue square, LacNAc-core, green circle, free-*N*-glycans, yellow square, free-mucin-type; orange triangle, Xyl-Glc-core; purple diamond, other sialylated glycans; light blue diamond, other hexuronylated glycans; symbol with red rim, glycans with α1,3/4-fucosylation. The glycans with sialyl Lewis A are surrounded by a red dotted line. The glycans containing 6-sulfoated Glc/GlcNAc are surrounded by a black dotted line.

(Figure S12)

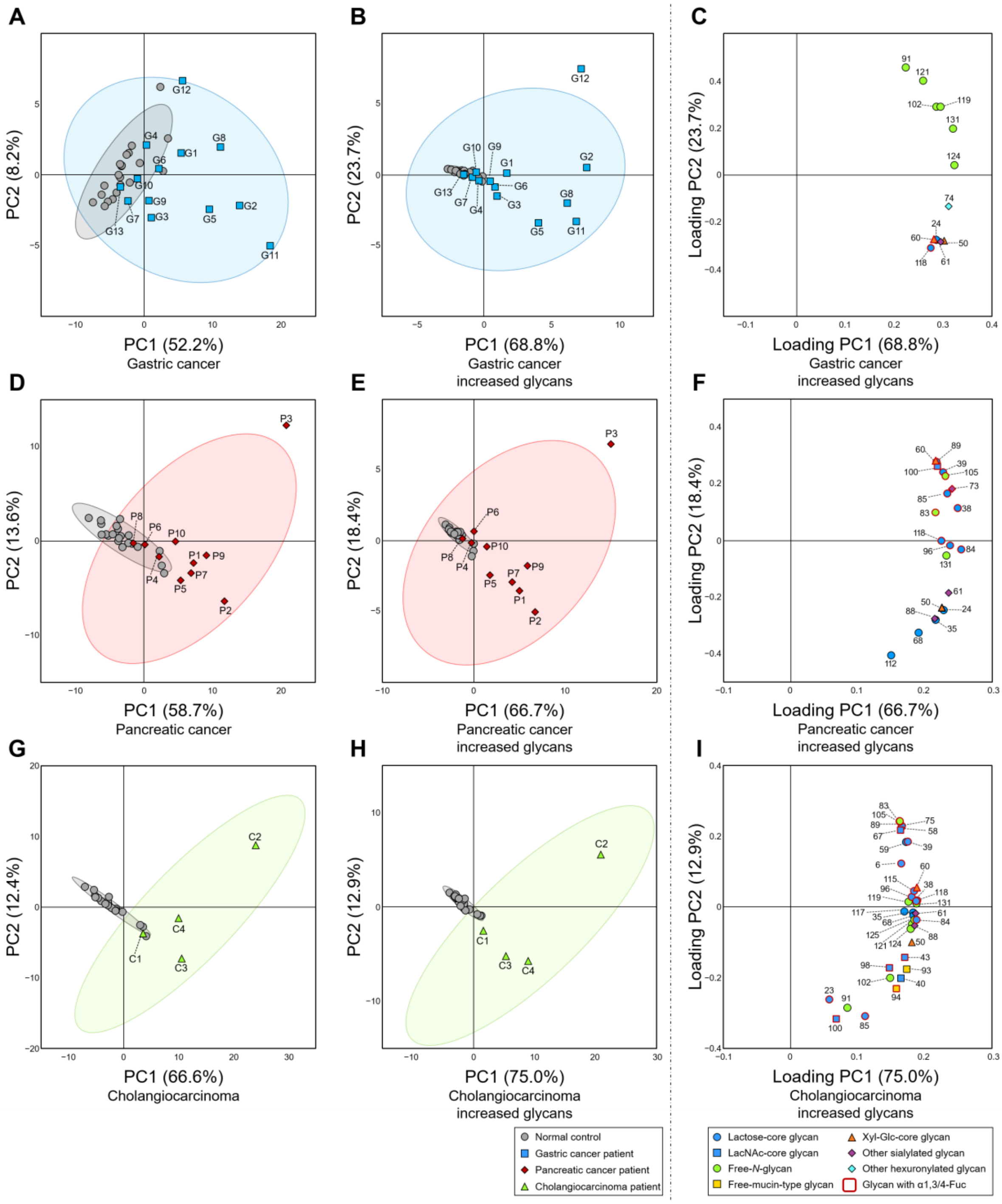

A
PC1 (52.2%)
Gastric cancer
PC2 (8.2%)
B
PC1 (68.8%)
Gastric cancer
increased glycans
PC2 (23.7%)
C
Loading PC1 (68.8%)
Gastric cancer
increased glycans
Loading PC2 (23.7%)
D
PC1 (58.7%)
Pancreatic cancer
PC2 (13.6%)
E
PC1 (66.7%)
Pancreatic cancer
increased glycans
PC2 (18.4%)
F
Loading PC1 (66.7%)
Pancreatic cancer
increased glycans
Loading PC2 (18.4%)
G
PC1 (66.6%)
Cholangiocarcinoma
PC2 (12.4%)
H
PC1 (75.0%)
Cholangiocarcinoma
increased glycans
PC2 (12.9%)
I
Loading PC1 (75.0%)
Cholangiocarcinoma
increased glycans
Loading PC2 (12.9%)
Normal control
Gastric cancer patient
Pancreatic cancer patient
Cholangiocarcinoma patient
Lactose-core glycan
LacNAc-core glycan
Free-N-glycan
Free-mucin-type glycan
Xyl-Glc-core glycan
Other sialylated glycan
Other hexuronylated glycan
Glycan with α1,3/4-Fuc

Fig. S12. Principal component analysis (PCA) of the glycans increased in cancer patients. PCA was performed using glycan levels obtained from SRM and fluorescent detection analysis (Figure 6, S9 and S10) in MetaboAnalyst 4.0 software. The plots were generated by Microsoft Excel 2013 for detailed labeling of the symbols. The data of each cancer patient group was separately subjected to PCA. (A–C) Gastric cancer patients. (D–F) Pancreatic cancer patients. (G–I) Cholangiocarcinoma patients. (A, D and G) PCA score plots of PC1 to PC2 using the data of the total analyzed glycans. (B, E and H) PCA score plots of PC1 to PC2 using the data of the glycans met the criteria for increased levels in each cancer patient group (Table SIV). (E and F) The PC2 axis shown is inverted. (H and I) Both the PC1 and the PC2 axes shown are inverted. (A, B, D, E, G and H) Symbols indicate the cases as follows: gray circle, normal control; blue square, gastric cancer patient; red diamond, pancreatic cancer patient; green triangle, cholangiocarcinoma patient. The ellipses correspond to 95% confidence region. (C, F and I) Loading plot of PC1 to PC2 for the score plots (B), (E) and (H), respectively. The glycans are plotted as symbols based on their structures, as follows; blue circle, lactose-core; blue square, LacNAc-core, green circle, free-*N*-glycans, yellow square, free-mucin-type; orange triangle, Xyl-Glc-core; purple diamond, other sialylated glycans; light blue diamond; other hexuronylated glycans.

Table SI. Clinical information of the normal controls and patients with gastric cancer, pancreatic cancer and cholangiocarcinoma (supplementary).

Case No. [a]	Sex	Age	CA19-9 (U/mL)[b]	CEA (ng/mL)[c]	ABO blood group	Urine Creatinine (mg/dL)	BUN[d] (mg/dL)	Serum Creatinine (mg/dL)[e]
N1	M	64	<2	nd[f]	O	61.6	13	1.14
N2	M	69	7	nd	O	175.0	18	0.80
N3	M	69	2	nd	A	59.8	11	0.86
N4	M	77	4	nd	A	190.6	22	0.82
N5	M	68	2	nd	O	116.7	15	1.02
N6	F	53	3	nd	AB	140.1	13	0.73
N7	F	70	3	nd	O	77.9	16	0.63
N8	M	75	14	nd	A	84.0	20	1.20
N9	M	81	2	nd	A	83.1	18	1.00
N10	M	50	12	nd	B	84.1	15	0.93
N11	F	75	2	nd	B	58.8	14	0.84
N12	M	69	4	nd	A	105.1	29	1.51
N13	F	33	5	nd	A	72.9	12	0.66
N14	F	56	2	nd	O	115.8	13	0.48
N15	M	81	4	nd	AB	80.6	16	0.85
N16	M	50	8	nd	A	281.4	15	1.01
N17	M	71	12	nd	A	43.8	15	0.71
N18	M	42	3	nd	A	94.2	21	0.90
N19	F	61	5	nd	B	77.2	14	0.60
N20	M	66	4	nd	A	126.9	17	0.83
N21	M	48	3	nd	A	148.4	12	0.74
G1	F	61	4	1.1	B	32.1	11	0.42
G2	F	65	67	1.0	A	384.7	11	0.69
G3	M	65	206	60.0	B	115.3	19	0.79
G4	M	78	568	6.2	AB	160.6	13	0.82
G5	F	70	<2	1.2	B	319.4	14	0.63
G6	M	64	4	7.5	O	190.4	17	1.04
G7	M	72	76	1.7	A	115.7	16	1.06
G8	M	74	3	1.1	A	186.4	42	1.65
G9	M	74	7	4.4	AB	62.5	17	1.02
G10	M	59	1063	27.4	A	28.6	8	0.79
G11	F	67	2	1.2	O	162.9	30	0.65
G12	F	62	16	549.9	O	187.1	13	0.59
G13	M	60	1187	4.5	A	254.3	20	0.92
P1	M	48	3311	3.7	A	69.4	12	0.72
P2	F	58	>100000	560.2	B	180.5	12	0.66
P3	M	68	>100000	220.5	A	439.5	18	0.86
P4	F	50	16421	11.5	A	50.5	8	0.59
P5	F	66	<2	3.0	B	159.5	14	0.69
P6	M	62	46597	4.8	A	270.1	16	0.97
P7	M	72	20124	13.1	A	51.5	15	0.48
P8	F	62	371	7.3	A	116.2	29	0.77
P9	M	64	>100000	162.9	O	187.7	15	0.86
P10	M	77	>100000	42.0	A	59.7	15	0.56
C1	F	55	29046	206.0	O	74.6	8	0.74
C2	M	65	32678	1673.1	AB	363.8	19	1.43
C3	F	78	>100000	164.4	O	45.4	15	0.93
C4	M	74	81803	156.2	O	153.4	13	0.81

(Table SI.)
a) G, P and C indicate gastric cancer, pancreatic cancer and cholangiocarcinoma, respectively.
b) Cut-off value of CA19-9 is 37 U/mL.
c) Cut-off values of CEA is 5 ng/mL.
d) Normal range of BUN is 8.0–20.0 mg/dL.
e) Normal range of serum creatinine (mg/dL) is 0.65–1.07 for male and 0.46–0.79 for female, respectively.
f) “nd” indicates “not determined”

Table SII. Estimated structures of acidic free-glycans from urine.

Glycan No. (#)[a]	Estimated structure[b]	Exp. *m/z*[c]	Theo. *m/z*	Ion species	Class
1	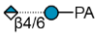	435.29	435.16	M+H	Other hexuronylated
2	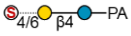	602.26	602.37	M+H+TEA	Lactose-core (small)
3	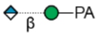	435.24	435.16	M+H	Other hexuronylated
4	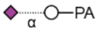	550.34	550.22	M+H	Other sialylated
5	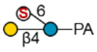	602.34	602.37	M+H+TEA	Lactose-core (small)
6	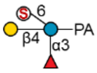	748.24	748.32	M+H+TEA	Lactose-core (small)
7	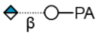	435.25	435.16	M+H	Other hexuronylated
8	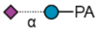	550.31	550.22	M+H	Other sialylated
9	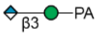	435.29	435.16	M+H	Other hexuronylated
10	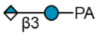	435.21	435.16	M+H	Other hexuronylated
11	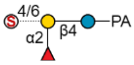	748.40	748.32	M+H+TEA	Lactose-core (small)
12	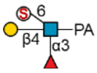	789.37	789.34	M+H+TEA	LacNAc-core (small)
13	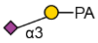	550.35	550.22	M+H	Other sialylated
14	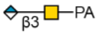	476.33	476.19	M+H	Other hexuronylated
15	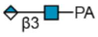	476.30	476.19	M+H	Other hexuronylated
16	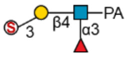	789.35	789.32	M+H+TEA	LacNAc-core (small)
17	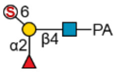	789.35	789.32	M+H+TEA	LacNAc-core (small)
18	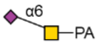	591.37	591.25	M+H	Free-mucin-type
19	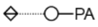	435.39	435.16	M+H	Other hexuronylated
20	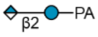	435.25	435.16	M+H	Other hexuronylated
21	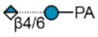	435.27	435.16	M+H	Other hexuronylated
22	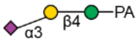	712.36	712.28	M+H	Lactose-core (small, C2-epimer)
23	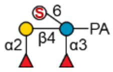	894.34	894.37	M+H+TEA	Lactose-core (small)
24	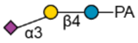	712.39	712.28	M+H	Lactose-core (small)
25	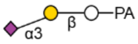	712.42	712.28	M+H	Other sialylated
26	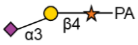	682.39	682.27	M+H	Other sialylated
27	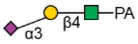	753.41	753.30	M+H	LacNAc-core (small, C2-epimer)
28	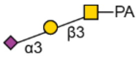	753.44	753.30	M+H	Free-mucin-type glycans
29	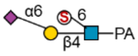	934.32	934.38	M+H+TEA	LacNAc-core (small)
30	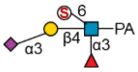	1080.34	1080.44	M+H+TEA	LacNAc-core (small)
31	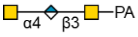	679.39	679.27	M+H	Other hexuronylated
32	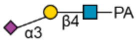	753.42	753.30	M+H	LacNAc-core (small)

33	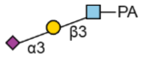	753.47	753.30	M+H	Free-mucin-type (C2-epimer)
34	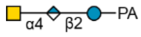	638.35	638.24	M+H	Other hexuronylated
35	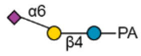	712.34	712.28	M+H	Lactose-core (small)
36	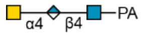	679.35	679.27	M+H	Other hexuronylated
37	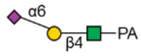	753.35	753.30	M+H	LacNAc-core (small, C2-epimer)
38	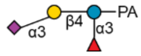	858.46	858.33	M+H	Lactose-core (small)
39	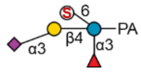	1039.48	1039.41	M+H+TEA	Lactose-core (small)
40	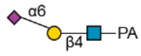	753.38	753.30	M+H	LacNAc-core (small)
41	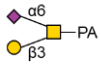	753.42	753.30	M+H	Free-mucin-type glycans
42	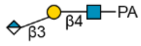	638.38	638.24	M+H	LacNAc-core (small)
43	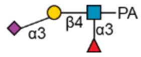	899.45	899.36	M+H	LacNAc-core (small)
44	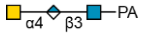	679.37	679.27	M+H	Other hexuronylated
45	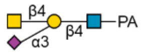	956.49	956.38	M+H	LacNAc-core (small)
46	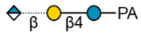	597.35	597.21	M+H	Lactose-core (small)
47	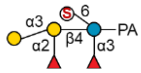	1056.33	1056.43	M+H+TEA	Lactose-core (small)
48	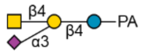	915.41	915.36	M+H	Lactose-core (small)
49	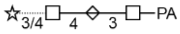	811.37	811.31	M+H	Other hexuronylated
50	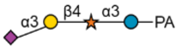	844.43	844.32	M+H	Xyl-Glc-core
51	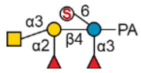	1097.38	1097.45	M+H+TEA	Lactose-core (small)
52	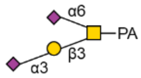	1044.42	1044.40	M+H	Free-mucin-type
53	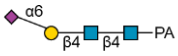	956.45	956.45	M+H	Free-*N*-glycans ($GlcNAc_2$)
54	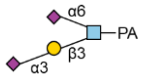	1044.44	1044.40	M+H	Free-mucin-type (C2-epimer)
55	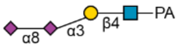	1044.52	1044.40	M+H	LacNAc-core (small)
56	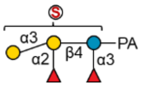	1056.37	1056.43	M+H+TEA	Lactose-core (small)
57	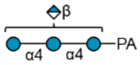	759.35	759.27	M+H	Other hexuronylated
58	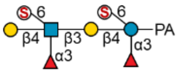	1440.28	1440.58	M+H+2TEA	Lactose-core (LacNAc-extended)
59	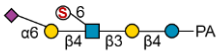	1258.35	1258.49	M+H+TEA	Lactose-core (LacNAc-extended)
60	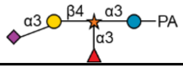	990.46	990.38	M+H	Xyl-Glc-core

61	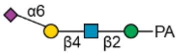	915.43	915.36	M+H	Other sialylated
62	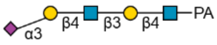	1118.50	1118.44	M+H	LacNAc-core (LacNAc-extended)
63	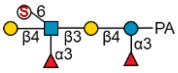	1259.41	1259.51	M+H+TEA	Lactose-core (LacNAc-extended)
64	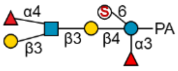	1259.45	1259.51	M+H+TEA	Lactose-core (LacNAc-extended)
65	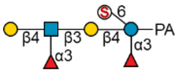	1259.39	1259.51	M+H+TEA	Lactose-core (LacNAc-extended)
66	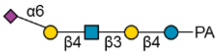	1077.50	1077.41	M+H	Lactose-core (LacNAc-extended)
67	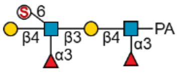	1300.32	1300.53	M+H+TEA	LacNAc-core (LacNAc-extended)
68	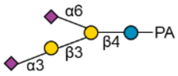	1165.41	1165.43	M+H	Lactose-core (small)
69	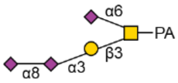	1335.46	1335.49	M+H	Free-mucin-type
70	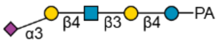	1077.50	1077.41	M+H	Lactose-core (LacNAc-extended)
71	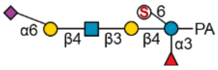	1404.32	1404.54	M+H+TEA	Lactose-core (LacNAc-extended)
72	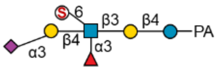	1404.41	1404.54	M+H+TEA	Lactose-core (LacNAc-extended)
73	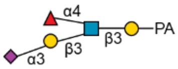	1061.47	1061.41	M+H	Other sialylated
74	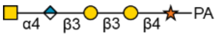	932.46	932.34	M+H	Other hexuronylated
75	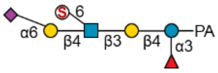	1404.42	1404.54	M+H+TEA	Lactose-core (LacNAc-extended)
76	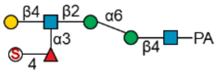	1316.38	1316.53	M+H+TEA	Free-*N*-glycans (GlcNAc$_1$)
77	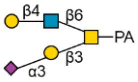	1118.48	1118.44	M+H	Free-mucin-type
78	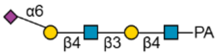	1118.65	1118.44	M+H	LacNAc-core (LacNAc-extended)
79	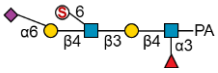	1445.45	1445.57	M+H+TEA	LacNAc-core (LacNAc-extended)
80	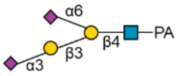	1206.52	1206.45	M+H	LacNAc-core (small)
81	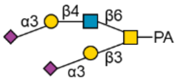	1409.72	1409.53	M+H	Free-mucin-type
82	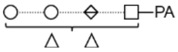	1092.57	1092.41	M+H	Other hexuronylated
83	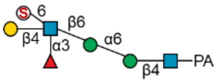	1316.41	1316.53	M+H+TEA	Free-*N*-glycans (GlcNAc$_1$)
84	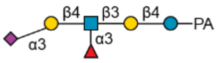	1223.48	1223.49	M+H	Lactose-core (LacNAc-extended)

85	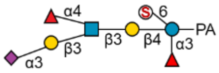	1550.61	1550.60	M+H+TEA	Lactose-core (LacNAc-extended)
86	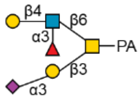	1264.69	1264.49	M+H	Free-mucin-type
87	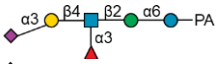	1223.73	1223.47	M+H	Other sialylated
88	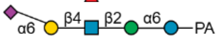	1077.61	1077.41	M+H	Other sialylated
89	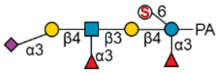	1550.62	1550.60	M+H+TEA	Lactose-core (LacNAc-extended)
90	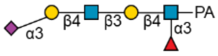	1264.56	1264.49	M+H	LacNAc-core (LacNAc-extended)
91	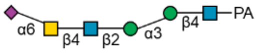	1321.64	1321.52	M+H	Free-*N*-glycans ($GlcNAc_1$)
92	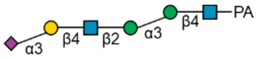	1280.68	1280.49	M+H	Free-*N*-glycans ($GlcNAc_1$)
93	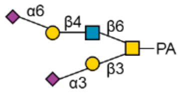	1409.63	1409.53	M+H	Free-mucin-type
94	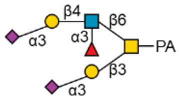	1555.71	1555.59	M+H	Free-mucin-type
95	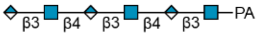	1234.42	1234.41	M+H	Other hexuronylated
96	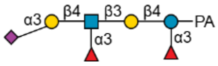	1369.56	1369.53	M+H	Lactose-core (LacNAc-extended)
97	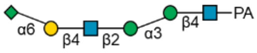	1239.41	1239.46	M+H	Free-*N*-glycans ($GlcNAc_1$)
98	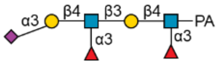	1410.60	1410.55	M+H	LacNAc-core (LacNAc-extended)
99	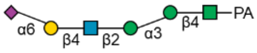	1280.57	1280.49	M+H	Free-*N*-glycans ($GlcNAc_1$, C2-epimer)
100	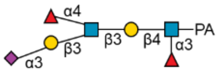	1410.59	1410.55	M+H	LacNAc-core (LacNAc-extended)
101	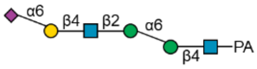	1280.73	1280.49	M+H	Free-*N*-glycans ($GlcNAc_1$)
102	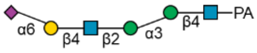	1280.49	1280.49	M+H	Free-*N*-glycans ($GlcNAc_1$)
103	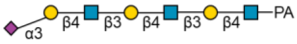	1483.52	1483.57	M+H	LacNAc-core (LacNAc-extended)
104	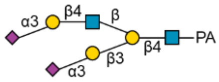	1571.56	1571.58	M+H	LacNAc-core (LacNAc-extended)
105	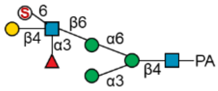	1478.44	1478.58	M+H+TEA	Free-*N*-glycans ($GlcNAc_1$)
106	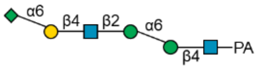	1239.50	1239.46	M+H	Free-*N*-glycans ($GlcNAc_1$)
107	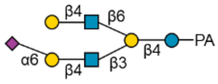	1442.51	1442.54	M+H	Lactose-core (LacNAc-extended)
108	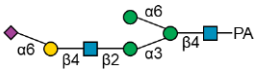	1442.17	1442.54	M+H	Free-*N*-glycans ($GlcNAc_1$)

109	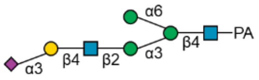	1442.91	1442.54	M+H	Free-*N*-glycans ($GlcNAc_1$)
110	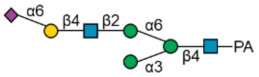	1442.31	1442.54	M+H	Free-*N*-glycans ($GlcNAc_1$)
111	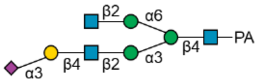	1645.36	1645.62	M+H	Free-*N*-glycans ($GlcNAc_1$)
112	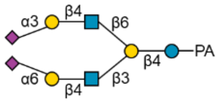	1733.83	1733.64	M+H	Lactose-core (LacNAc-extended)
113	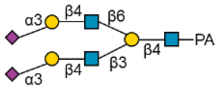	1774.67	1774.66	M+H	LacNAc-core (LacNAc-extended)
114	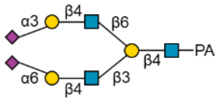	1774.67	1774.66	M+H	LacNAc-core (LacNAc-extended)
115	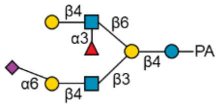	1588.32	1588.59	M+H	Lactose-core (LacNAc-extended)
116	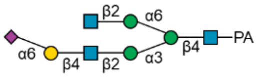	1645.32	1645.62	M+H	Free-*N*-glycans ($GlcNAc_1$)
117	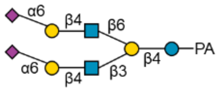	1733.82	1733.64	M+H	Lactose-core (LacNAc-extended)
118	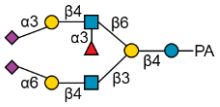	1879.37	1879.69	M+H	Lactose-core (LacNAc-extended)
119	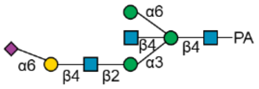	1645.39	1645.62	M+H	Free-*N*-glycans ($GlcNAc_1$)
120	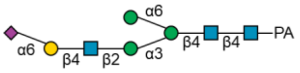	1645.36	1645.62	M+H	Free-*N*-glycans ($GlcNAc_2$)
121	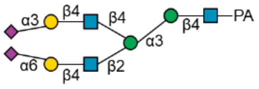	1936.39	1936.72	M+H	Free-*N*-glycans ($GlcNAc_1$)
122	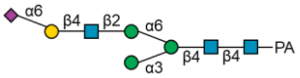	1645.34	1645.62	M+H	Free-*N*-glycans ($GlcNAc_2$)
123	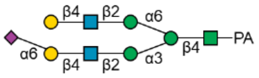	1807.32	1807.67	M+H	Free-*N*-glycans ($GlcNAc_1$, C2-epimer)
124	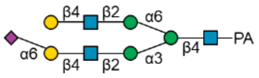	1807.35	1807.67	M+H	Free-*N*-glycans ($GlcNAc_1$)
125	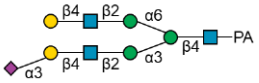	1807.30	1807.67	M+H	Free-*N*-glycans ($GlcNAc_1$)
126	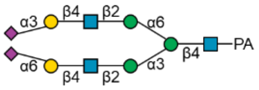	2098.38	2098.77	M+H	Free-*N*-glycans ($GlcNAc_1$)
127	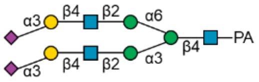	2098.31	2098.77	M+H	Free-*N*-glycans ($GlcNAc_1$)

128	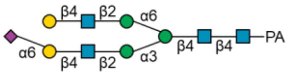	2010.74	2010.75	M+H	Free-*N*-glycans ($GlcNAc_2$)
129	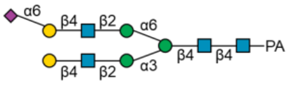	2010.62	2010.75	M+H	Free-*N*-glycans ($GlcNAc_2$)
130	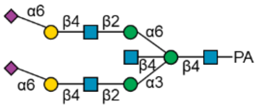	2301.58	2301.85	M+H	Free-*N*-glycans ($GlcNAc_1$)
131	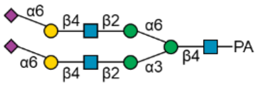	2098.23	2098.77	M+H	Free-*N*-glycans ($GlcNAc_1$)
132	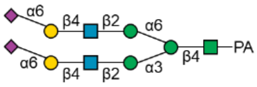	2098.43	2098.77	M+H	Free-*N*-glycans ($GlcNAc_1$, C2-epimer)
133	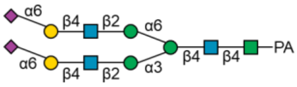	2301.69	2301.85	M+H	Free-*N*-glycans ($GlcNAc_2$, C2-epimer)
134	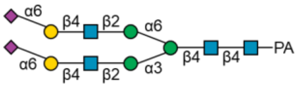	2301.73	2301.85	M+H	Free-*N*-glycans ($GlcNAc_2$)
135	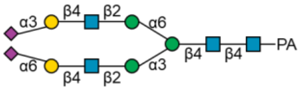	2301.95	2301.85	M+H	Free-*N*-glycans ($GlcNAc_2$)

a) Glycan numbers are from Figure 3.
b) Monosaccharide symbols are according to the symbol nomenclature for glycans, and indicated as follows: blue circle, Glc; blue square, GlcNAc; green circle, Man; green square, ManNAc; yellow circle, Gal; yellow square, GalNAc; light-blue square, TalNAc; red triangle, Fuc; purple diamond, Sialic acid (NeuAc); green diamond, KDN; divided diamond with blue upper side and white lower side, GlcA; white circle, unassigned Hex; white square, unassigned HexNAc; white triangle, unassigned dHex; white divided diamond, unassigned HexA; circled “S”, sulfate.
c) Representative data are shown.

Table SIII. Q1 and Q3 values for SRM measurements of the PA-glycans.

Glycan no. (#)	Q1 (*m/z*)	Q3[a] (*m/z*)		Glycan no. (#)	Q1 (*m/z*)	Q3[a] (*m/z*)	
Lactose-core glycans				Free-*N*-glycans			
6	748.3	**567.2**	421.2	76	1316.5	**1135.5**	300.2
11	748.3	**567.2**	259.1	83	1316.5	**624.3**	1135.5
23	894.4	**567.2**	713.3	91	1321.5	**1030.4**	300.2
24	712.3	**421.2**	259.1	92	1280.5	**300.2**	989.4
35	712.3	**421.2**	259.1	102	1280.5	**300.2**	989.4
38	858.3	**567.2**	712.3	105	1478.6	**1297.5**	786.3
39	1039.4	**858.3**	712.3	106	1239.5	**989.4**	300.2
58	1440.6	**1259.5**	259.1	108	1442.5	**300.2**	989.4
59	1258.5	**421.2**	1077.4	119	1645.6	**1354.5**	300.2
66	1077.4	**786.3**	421.2	120	1645.6	**1354.5**	300.2
68	1165.4	**874.3**	421.2	121	1936.7	**1645.6**	300.2
70	1077.4	**259.1**	421.2	124	1807.7	**1516.6**	989.4
71, 72	1404.5	**1223.5**	932.4	125	1807.7	**1516.6**	300.2
75	1404.5	**1223.5**	259.1				
84	1223.5	**421.2**	932.4	Free-mucin-type glycans			
85	1550.6	**1369.5**	259.1	28	753.3	**300.2**	462.2
89	1550.6	**1369.5**	259.1	52	1044.4	**462.2**	753.3
96	1369.5	**421.2**	259.1	81	1409.5	**753.3**	300.2
112	1733.6	**259.1**	932.4	93	1409.5	**300.2**	753.3
115	1588.6	**1297.5**	1077.4	94	1555.6	**753.3**	300.2
117	1733.6	**1151.4**	1077.4				
118	1879.7	**1077.4**	932.4	Xyl-Glc-core glycans			
				50	844.3	**259.1**	553.2
LacNAc-core glycans				60	990.4	**259.1**	699.3
12	789.3	**608.3**	462.2				
16	789.3	**608.3**	462.2	Other sialylated glycans			
29	934.4	**753.3**	462.2	13	550.2	**259.1**	
30	1080.4	**899.4**	608.3	61	915.4	**259.1**	624.3
32	753.3	**300.2**	462.2	74	1061.4	**259.1**	770.3
40	753.3	**462.2**	300.2	88	1077.4	**786.3**	421.2
43	899.4	**608.3**	300.2				
55	1044.4	**753.3**	462.2	Other hexuronylated glycans			
62	1118.4	**300.2**	827.3	10	435.2	**259.1**	
67	1300.5	**300.2**	1119.5	15	476.2	**300.2**	
98	1410.6	**300.2**	973.4	34	638.2	**435.2**	259.1
100	1410.6	**973.4**	300.2	36	679.3	**476.2**	300.2
113, 114	1774.7	**1118.4**	300.2	44	679.3	**476.2**	300.2
				73	932.3	**229.1**	553.2
				95	1234.4	**476.2**	300.2

a) Two transitions were measured except for the disaccharides. Only the transition shown in bold was used as the quantifier transition.

Table SIV. Comparison between cancer patient groups and normal controls for each glycan level.

Glycan no. (#)	Estimated Structures	Gastric cancer *p*-values[a]	Gastric cancer Fold change[b]	Pancreatic cancer *p*-values[a]	Pancreatic cancer Fold change[b]	Cholangiocacinoma *p*-values[a]	Cholangiocacinoma Fold change[b]
6	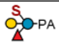	0.2869	0.8	0.0793	2.2	0.0307	3.9
10	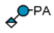	0.6633	0.9	0.0277	2.3	0.4515	1.3
11	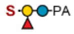	0.2751	1.1	0.3469	1.1	0.0191	2.3
12	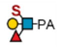	0.5261	1.1	0.5172	1.2	0.0112	2.5
13	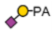	< 0.0001	2.8	< 0.0001	2.7	0.0003	2.6
15	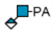	0.0422	1.3	0.0022	1.5	0.0149	1.7
16	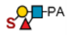	0.9943	1.0	0.8005	1.0	0.0245	2.1
23	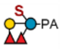	0.4330	0.6	0.5725	1.1	0.0149	4.8
24	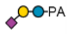	< 0.0001	3.4	< 0.0001	4.7	0.0011	2.9
28	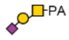	0.0002	1.9	< 0.0001	2.0	0.0003	2.4
29	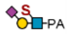	0.1197	1.2	0.4219	1.2	0.0568	2.4
30	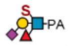	0.0046	1.3	0.0116	1.7	0.0028	2.5
32	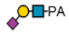	0.0003	1.8	0.0009	1.8	0.0011	2.4
34	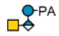	0.2174	1.3	< 0.0001	2.5	0.1537	1.7
35	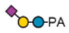	0.0005	2.8	< 0.0001	3.8	0.0019	3.2
36		0.0052	1.6	< 0.0001	1.7	0.0028	1.6
38	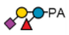	0.0116	2.1	0.0003	5.5	0.0003	6.9
39	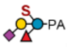	0.6968	1.2	0.0042	6.3	0.0011	18.0
40	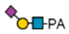	< 0.0001	2.8	< 0.0001	2.7	0.0011	3.0
43	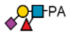	< 0.0001	2.6	< 0.0001	2.7	0.0002	4.1
44	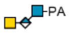	0.0066	1.4	0.0049	1.3	0.0112	1.6
50	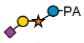	< 0.0001	3.4	< 0.0001	4.1	0.0002	3.6
52	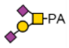	0.0016	1.6	0.0004	1.7	0.0006	1.9
55	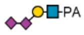	0.5156	1.3	0.5376	1.2	0.1220	1.8
58	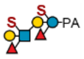	0.7333	0.8	0.2913	1.9	0.0145	21.6
59	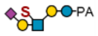	0.0265	1.4	0.0132	2.1	0.0381	4.0
60	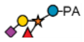	< 0.0001	5.2	< 0.0001	10.4	0.0002	13.9
61	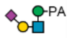	< 0.0001	3.2	< 0.0001	3.0	0.0019	3.5
62	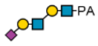	0.0016	1.6	0.0031	1.5	0.0006	2.0
66	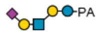	0.2451	1.8	0.0004	2.7	0.1134	2.9
67	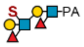	0.5816	0.9	0.0534	2.6	0.0191	7.0
68	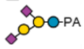	0.0003	2.8	< 0.0001	4.4	0.0019	3.8
70	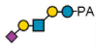	0.0647	1.4	0.0011	2.8	0.2032	1.4
71,72	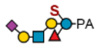 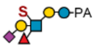	0.5422	0.9	0.2008	2.4	0.0814	4.2
73	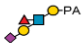	0.5115	2.0	0.0171	5.9	0.0149	2.7
74	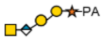	0.0002	3.2	< 0.0001	1.8	0.0002	2.1
75	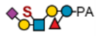	0.0018	1.0	0.0004	2.6	0.0084	25.2
76	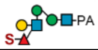	< 0.0001	1.6	< 0.0001	1.6	0.0019	1.7

81	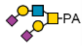	< 0.0001	2.5	< 0.0001	2.0	0.0002	2.3
83	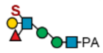	0.0002	2.5	< 0.0001	3.0	0.0006	37.0
84	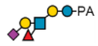	0.7936	3.5	0.0277	3.6	0.0084	4.0
85	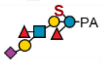	0.0002	0.9	< 0.0001	6.1	0.0011	3.4
88	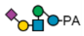	0.2910	3.1	0.0088	3.8	0.0245	3.8
89	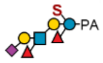	0.0012	1.2	0.0008	5.4	0.0003	102.5
91	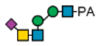	0.0130	5.6	0.0016	2.0	0.0003	5.6
92	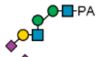	0.0006	2.1	0.0008	1.6	0.0002	2.6
93	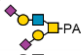	0.0021	2.2	0.0003	2.0	0.0002	3.2
94	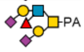	0.0006	2.1	< 0.0001	2.3	0.0006	3.5
95	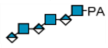	0.1034	1.6	0.0036	2.0	0.0060	2.0
96	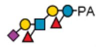	0.0040	1.8	0.0022	3.6	0.0019	3.6
98	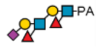	> 0.9999	1.8	0.0001	2.2	0.0009	4.6
100	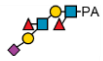	0.0001	1.0	< 0.0001	29.0	0.0002	13.3
102	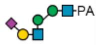	< 0.0001	6.0	< 0.0001	2.5	0.0002	5.5
105	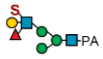	0.0144	2.6	0.0019	4.3	0.0307	60.2
106	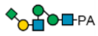	< 0.0001	1.8	< 0.0001	1.6	0.0003	1.8
108	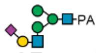	0.2309	2.7	0.0004	2.4	0.4515	2.8
112	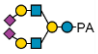	0.0008	1.4	0.0003	3.0	0.0043	1.6
113,114	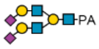 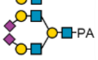	< 0.0001	1.9	0.0066	2.1	0.0149	2.4
115	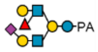	0.0027	2.8	0.0009	2.9	0.0060	5.9
117	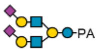	0.0292	2.1	0.0001	2.9	0.0112	3.2
118	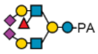	< 0.0001	3.6	0.0002	3.7	0.0019	4.5
119	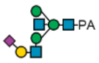	< 0.0001	5.6	0.0001	2.4	0.0002	4.7
120	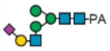	< 0.0001	2.3	< 0.0001	2.0	0.0003	2.5
121	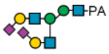	0.0005	4.6	< 0.0001	2.6	0.0002	4.5
124	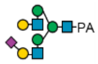	< 0.0001	3.0	< 0.0001	2.5	0.0002	5.3
125	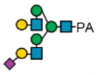	< 0.0001	2.7	0.0002	2.1	0.0019	4.3
131	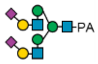	< 0.0001	4.7	< 0.0001	3.0	0.0002	7.1
134	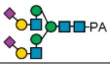	0.0002	2.7	< 0.0001	2.2	0.0002	2.6

Related to Figure 6, S9 and S10, the glycan levels of the groups of patients with gastric cancer (n=13), pancreatic cancer (n=10) and cholangiocarcinoma (n=4) and normal controls (n=21) are compared. In this table, the number and values of the glycans that meet simultaneously p-value < 0.05 and fold change ≥ 3.0 are labeled with an orange background.

a) The p-values were calculated by Mann−Whitney U test.

b) The ratio of mean values of each cancer group to normal controls.
